# High-efficiency magnetophoretic labelling of adoptively-transferred T cells for longitudinal *in vivo* Magnetic Particle Imaging

**DOI:** 10.7150/thno.95527

**Published:** 2024-09-23

**Authors:** Rong En Tay, Lokamitra P, Shun Toll Pang, Kay En Low, Hui Chien Tay, Charmaine Min Ho, Benoit Malleret, Olaf Rötzschke, Malini Olivo, Zhi Wei Tay

**Affiliations:** 1 Singapore Immunology Network (SIgN), Agency for Science, Technology and Research (A*STAR), 8A Biomedical Grove, #04-06 Immunos, Singapore 138648, Republic of Singapore.; 2 Institute of Bioengineering and Bioimaging (IBB), Agency for Science, Technology and Research (A*STAR), 11 Biopolis Way, #02-02 Helios Building, Singapore 138667, Republic of Singapore.; 3 Electron Microscopy Unit, Yong Loo Lin School of Medicine, National University of Singapore, Singapore 117597, Republic of Singapore.; 4 Department of Microbiology and Immunology, Immunology Translational Research Programme, Yong Loo Lin School of Medicine, National University of Singapore, Republic of Singapore.; 5 School of Biological Sciences, Nanyang Technological University, 50 Nanyang Avenue, Singapore 639798, Republic of Singapore.; 6 A*STAR Skin Research Labs (A*SRL), Agency for Science, Technology and Research (A*STAR), 31 Biopolis Way, #07-01 Nanos, Singapore 138669, Republic of Singapore.; 7 National Institute of Advanced Industrial Science and Technology (AIST), Health and Medical Research Institute (HMRI), 1-2-1 Namiki, Tsukuba, Ibaraki 305-8564, Japan.

## Abstract

While adoptive cell therapies (ACT) have been successful as therapies for blood cancers, they have limited efficacy in treating solid tumours, where the tumour microenvironment excludes and suppresses adoptively transferred tumour-specific immune cells. A major obstacle to improving cell therapies for solid tumours is a lack of accessible and quantitative imaging modalities capable of tracking the migration and immune functional activity of ACT products for an extended duration *in vivo*.

**Methods:** A high-efficiency magnetophoretic method was developed for facile magnetic labelling of hard-to-label immune cells, which were then injected into tumour-bearing mice and imaged over two weeks with a compact benchtop Magnetic Particle Imager (MPI) design.

**Results:** Labelling efficiency was improved more than 10-fold over prior studies enabling longer-term tracking for at least two weeks *in vivo* of the labelled immune cells and their biodistribution relative to the tumour. The new imager showed 5-fold improved throughput enabling much larger density of data (up to 20 mice per experiment).

**Conclusions:** Taken together, our innovations enable the convenient and practical use of MPI to visualise the localisation of ACT products in *in vivo* preclinical models for longitudinal, non-invasive functional evaluation of therapeutic efficacy.

## Introduction

In the last decade, adoptive cell therapies (ACT) for cancer have achieved remarkable success in treating haematological malignancies 1-4. However, unlike in haematological cancers where the cell therapy product has ready access to its intended target of cancer cells, the complex heterogeneous microenvironment of solid tumours both suppresses and physically excludes anti-tumour effector immune cells from entering the tumour site 5,6. The current challenge in rational design and engineering of ACTs to circumvent these obstacles is the lack of knowledge of intrinsic biological factors that drive successful or unsuccessful responses to cell therapy. This is compounded by a lack of practical, non-invasive methods to track the performance of the adoptively transferred cell therapy product across the duration of treatment. Similarly, the experience gained from clinical trials of ACTs for solid tumours thus far highlights the increasing need for real-time information on the biodistribution of ACT products in patients for timely therapy adjustments to achieve treatment success and to avoid immune-related adverse events (irAEs) 1,2. Specifically, the key information sought by both preclinical researchers and clinicians are: (1) where adoptively transferred cells migrate and localise to; and (2) whether the patterns of their trafficking correlate with outcomes of treatment and/or disease progression.

To address these needs for real-time, non-invasive longitudinal tracking of cell therapy products, imaging is the best suited approach. Magnetic Resonance Imaging (MRI), ultrasound imaging, optical imaging (e.g. fluorescence- or bioluminescence-imaging, FLI and BLI), and nuclear imaging methods (e.g. positron emission tomography, PET) are four broad categories of existing methods for *in vivo* imaging with different performance characteristics, strengths, and limitations. However, none of them are ideally suited for longitudinal imaging of adoptively transferred cells. MRI has long-lasting contrast agents but these are only semi-quantitative and work by generating hypointense image spots that may be difficult to distinguish from naturally hypointense anatomy (e.g. lungs, tendon, bones, or air interfaces) 7,8. Moreover, MRI systems are relatively costly, large, and require dedicated technical specialists to operate. Ultrasound is relatively inexpensive and portable, but it does not work in acoustically shadowed body regions and image quality is highly dependent on operator proficiency 9,10. Next, while FLI and BLI are commonly used to image cells *in vivo* in preclinical model systems, their range of clinical applications is limited by the shallow depth of light penetration into human tissue 11. In addition, FLI and BLI often require genetic modifications to cells of interest (e.g. expression of a fluorescent or bioluminescent protein reporter), which precludes their use in clinical settings. Finally, nuclear imaging techniques are highly sensitive, but are less practical for longitudinal tracking studies due to the very short tracer lifetime (the most commonly used ^18^F isotope has half-life of 2 h) 12 and carry the increased regulatory and safety requirements for working with radioactive material.

In comparison, magnetic particle imaging (MPI) is an emerging imaging technique first invented in 2005 by Gleich and Weizenecker 13, and it has recently taken large strides towards clinical translation 14-16. Magnetic particle imaging (MPI) technology works by imaging electronic superparamagnetism, which is up to 22 million times stronger than the ^1^H nuclear paramagnetism imaged by MRI 17, enabling sensitive tracking of superparamagnetic “tracers”. In terms of imaging principles, MPI has two key advantages over the four existing imaging modalities. First, MPI signal is linearly proportional to the mass of “tracer” iron label present, enabling fully quantitative imaging while being long-lasting and non-radioactive 18,19. Second, MPI signal does not suffer from attenuation *in vivo* as the very-low-frequency magnetic fields used in MPI are not generated or affected by biological tissue 20. This imparts ideal contrast and permits imaging of any anatomical region without the view limits of optical or ultrasound techniques 21,22. In addition, MPI's iron oxide labels are known to be safe, having been already approved as clinical imaging agents (i.e. Feraheme^TM^ (ferumoxytol), Resovist^TM^ (ferucarbotran), and Resotran^TM^ (ferucarbotran)) 23-27, and can be easily functionalised for specific biological purposes 28. Altogether, these characteristics make MPI a uniquely suited imaging modality for longitudinal cell tracking in general which has been pursued for 15 years since the invention of MPI in 2005. These early studies demonstrated *in vivo* tracking of stem cells 18,22,29 and labelling red blood cells for blood pool imaging 30. More recently in 2021, the first MPI tracking studies of T lymphocytes *in vivo* were reported 31,32.

However, the widespread use of MPI for both preclinical and clinical imaging of labelled cells of interest has been limited by two important factors - (1) the wide variability of permissivity to labelling across cell types compounded by the lack of robust methods to label non-permissive (refractory) cell types with sufficient MNPs to sustain long-term imaging, and (2) the relatively higher cost and complexity of existing commercial MPI scanners that utilise an MRI-like, in-bore design based on high-power and costly electromagnetic gradient coils 33-35.

Here we directly addressed these two challenges by (1) using well-established magnetofection concepts 36 to develop a non-oscillatory, point-focal magnetophoretic method to significantly increase MNP uptake in hard-to-label T lymphocytes without the use of chemical vehicles (this is the first reported MPI usage of magnetofection for improving labelling for an *in vivo* cell tracking study), and (2) by engineering a MPI imaging platform that meets the sensitivity, linearity, and resolution requirements for convenient, high-throughput imaging of mice. Our results show that the new magnetophoretic method demonstrates a 3-fold increase in labelling on top of the increase from nanoparticle surface functionalisation with cell penetrating peptides (CPPs) for murine naïve CD8 T cells, which are difficult to label due to their small cytoplasmic volume and quiescent state. By combining these two innovations in our MPI workflow, we could achieve quantitative longitudinal imaging of the dynamics of antigen-specific CD8 T cell trafficking in the setting of immunisation for up to two weeks. We further demonstrated a potential application of MPI imaging in a preclinical model of ACT for solid tumours by using MPI to track the specific localisation of tumour antigen-specific CD8 T cells to antigen-positive tumours but not antigen-negative tumours within the same host mouse.

## Results

### A custom-designed magnetic plate allows efficient MNP labelling of hard-to-label immune cell types

**Figure 1:**
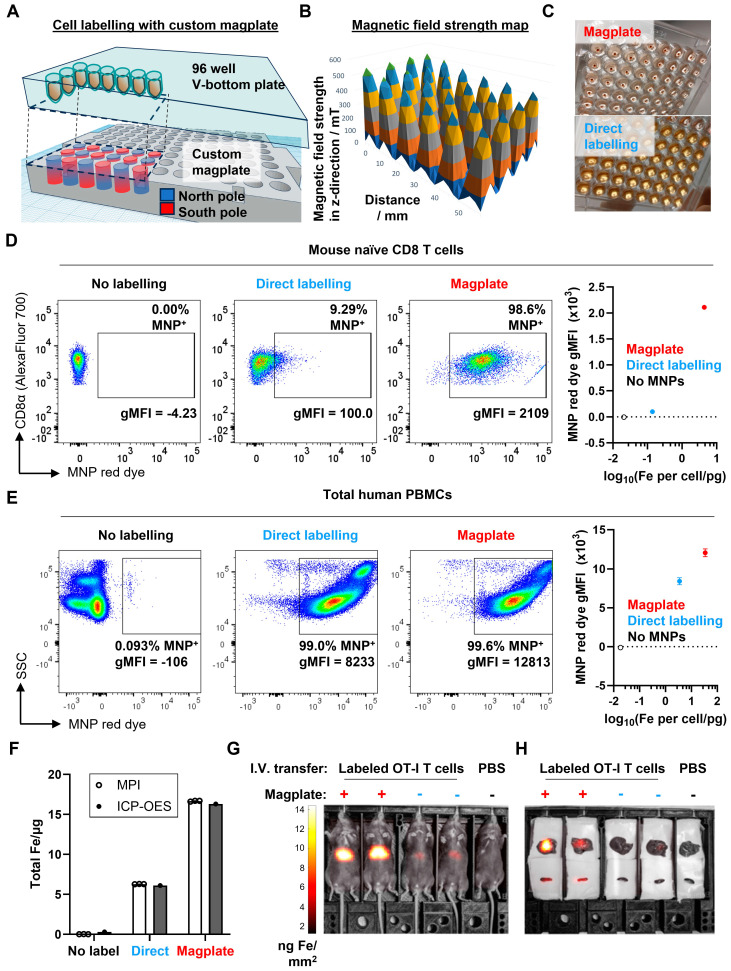
** Increased efficiency and faster kinetics of magplate MNP labelling over conventional direct labelling method. (A)** Illustration of the internal array of permanent NdFeB magnets embedded within our custom magplate showing the polarity of the magnets and their alignment below individual wells of a standard 96-well cell culture plate. **(B)** Mapping of the magnetic field strength along the vertical z-axis just above the surface of our custom magplate using a vertical-axis gaussmeter. **(C)** Representative images of primary naïve mouse CD8 T cells following 3 h of incubation with fluorescent MNPs in 96-well plates with (top) or without magplate incubation (bottom). **(D and E)** Representative flow cytometry plots (left) and quantitation (right) of MNP label uptake of mouse naïve CD8 T cells **(D)** and healthy human donor PBMCs **(E)** following 3 h of incubation under the indicated conditions. Iron uptake was quantified using MPI probe measurements. Data are representative of at least two independent experiments. **(F)** Total iron in cell pellets of equal cell numbers for unlabelled, direct-incubation labelled and magplate labelled experimental groups as in **(D)**, measured by MPI and ICP-OES methods **(G)** MPI *in vivo* image of healthy C57BL/6 at 24 h after intravenous transfer of OT-I CD8 T cells that were either MNP-labelled with the magplate method, direct-incubation method or unlabelled. **(H)** *Ex vivo* MPI scans of the livers (top row) and spleens (bottom row) of the mice from **(G)**.

To develop MPI imaging as an effective tool for the *in vivo* imaging of labelled cell populations, we first had to tackle the challenge of efficiently labelling hard-to-label immune cell types, such as naïve T cells. We designed a custom magplate comprising 96 individual neodymium-iron-boron alloy (NdFeB) permanent magnets embedded in alternating orientations within a plastic housing aligned exactly to the wells of a standard 96-well V-bottom plate (Figure 1A). The resulting magnetic field projected is focused upwards into the volume space of the plate wells (Figure 1B). Most prior magnetofection methods exert a magnetic force typically aligned along a single axis (vertically downwards) 36, generated with either a static single large magnet, a single electro-magnetic coil, or oscillatory magnetic fields generated by single electromagnets or the mechanical motion of permanent magnets. Our design presented here utilises a large number of arrayed small permanent magnets, each smaller than the well diameter of the 96-well plate, to exert static magnetic force in 3 axes.

Besides the vertical force drawing MNPs downwards, the horizontal forces have the added effect of focusing the MNPs into a narrower region within the horizontal plane, aligning the MNPs with the cell pellet at the narrow point of the V-bottom wells.

We next optimised a labelling protocol (see Methods) that would allow us to efficiently label even hard-to-label immune cells with cell penetrating peptide (CPP)-conjugated 70 nm MNPs within 3 h while preserving cell viability. After labelling, magplate-labelled immune cells formed a brown-coloured pellet at the bottom of the V-shaped wells with a clear supernatant (Figure 1C, top panel), whereas a parallel sample of the same immune cells labelled directly by co-incubation with MNPs appeared as off-white pellets while the supernatant remained turbid and brown (Figure 1C, bottom panel). Because the MNPs used were pre-conjugated with a fluorescent red dye, we also used flow cytometry to measure the shifts in red dye fluorescence resulting from MNP labelling of the immune cells. We found that the magplate labelling procedure resulted in almost complete labelling of hard-to-label naïve mouse CD8 T cells in 3 h, with a 10-fold increase in the percentage of label-positive cells and more than a 20-fold increase in geometric mean fluorescence intensity (gMFI) over cells that were directly labelled with CPP-conjugated MNPs for the same duration (Figure 1D). In contrast, we found that human PBMCs (hPBMCs) were relatively more permissive to labelling as even direct labelling resulted in almost complete labelling with MNPs. Nonetheless, using the magplate procedure to label hPBMCs increased the MNP label uptake by about 50% (as measured by red dye gMFI) while retaining at least the same efficiency of labelling (Figure 1E). Further analysis of MNP labelling efficiency in healthy donor PBMCs showed a general trend of increased MNP red dye gMFI across all 5 major immune subsets (monocytes, CD8 and CD4 T cells, B cells, and NK cells) when using the magplate labelling procedure over direct labelling (Figure S1). We further verified our results by directly measuring the total iron content of thoroughly washed Jurkat cell pellets (a human T cell-like cell line) after MNP labelling with and without the magplate using inductively coupled plasma-optical emission spectroscopy (ICP-OES), and by calculating iron content values from MPI measurements. The results from both methods showed that the magplate-labelled cell pellets contained more iron than the cell pellets from the directly labelled samples (Figure 1F).

Next, to test the durability of the cellular MNP label in an *in vivo* setting, we adoptively transferred 2 x 10^6^ magplate- or directly labelled naïve mouse OT-I CD8 T cells into healthy recipient mice and tracked the MNP label signal by total body MPI imaging over a period of 5 days. Consistent with the higher label uptake resulting from magplate labelling, we could detect clear MNP signal in the mice that received magplate-labelled OT-I cells (>10 ng Fe/mm^2^ in liver ROI), whereas the signal in mice that received non-magplate labelled OT-I cells was only about half as strong (between 4-6 ng Fe/mm^2^ in liver ROI) (Figure 1G). Importantly, when we performed *ex vivo* MPI imaging on freshly harvested livers and spleens 5 days after adoptive transfer of labelled cells, we could detect MNP iron signal in the spleens of mice that received magplate-labelled cells but could not distinguish MNP iron signal from the noise threshold in the spleens of mice that received non-magplate labelled cells (Figure 1H).

**Figure 2:**
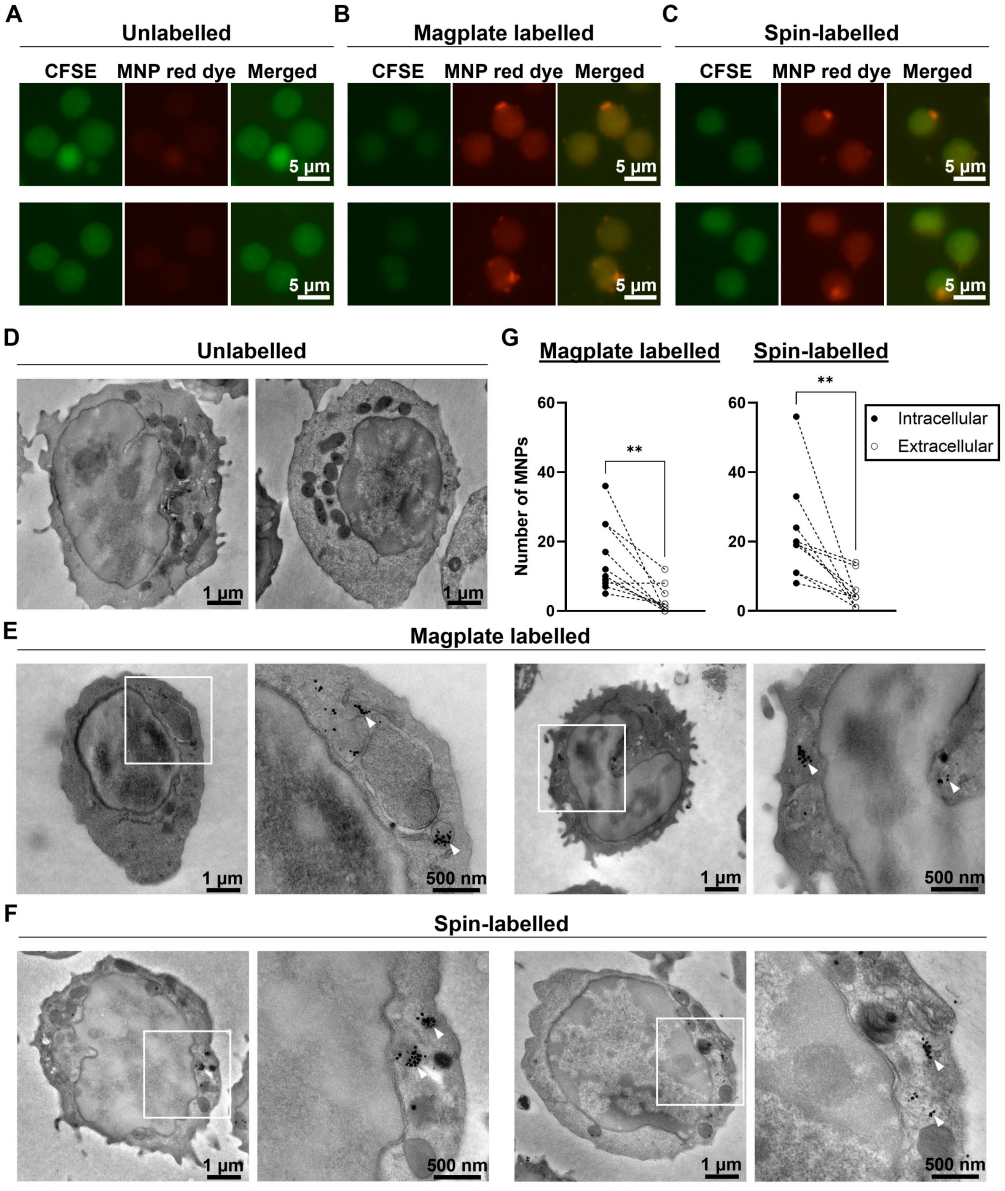
** Magplate labelling results in intracellular uptake of MNP labels. (A-C)** Representative live cell fluorescent images of primary mouse OT-I CD8 T cells labelled with cytosolic CFSE (green channel) prior to indicated labelling procedures. **(D-F)** Representative transmission electron microscope images of primary mouse OT-I CD8 T cells treated with indicated labelling procedures. White squares indicate regions of interest displayed at higher magnification to the right of whole cell image **(E and F)**. White arrowheads indicate MNPs in high-magnification images (**E and F**). **(G)** Quantification of intracellular and extracellular MNPs in images of cells from magplate- and spin-labelled samples (10 cells each). Statistical analysis using two-tailed paired-t test, **p < 0.01.

Finally, we investigated the localisation of the MNPs within labelled cells to confirm that the magplate labelling procedure indeed resulted in intracellular label uptake by the cells. We labelled naïve OT-I cells by magplate labelling and visualised the MNP label uptake by live cell fluorescent microscopy and transmission electron microscopy (TEM) imaging. As controls, we also imaged unlabelled cells and cells labelled by centrifugation (spin-labelled cells) as an alternative approach to labelling (previously reported here 37). For our live cell microscopy work, we labelled the cytosol of OT-I cells using carboxyfluorescein succinimidyl ester (CFSE) prior to MNP labelling to visualise the cells using fluorescent microscopy. After the labelling procedures, we observed the presence of red dye fluorescence within cells in magplate- and spin-labelled samples, but not within the intracellular regions of unlabelled cells (Figure 2A-C, Figure S2A-C). We also observed puncta of red dye fluorescence within some of the labelled cells (Figure 2B and 2C, Figure S2B and S2C).

Using parallel samples of OT-I cells from the same mice, we also performed TEM imaging to detect the presence of MNPs in labelled cells. We observed dark puncta indicating the presence of the electron-dense MNPs within the cytoplasm of magplate- and spin-labelled cells, but not within the cytoplasm of unlabelled cells (Figure 2D-F, Figure S2D-F). These dark puncta were often found clustered together in vesicle-like compartments within the cell bodies (Figure 2D-F, Figure S2D-F, panel insets). We further quantified the numbers of intracellular and extracellular MNPs (MNPs adhering to the outer leaflet of the plasma membrane) in at least 10 cells from each sample.

We found that there was a significant difference in the numbers of intracellular and extracellular MNPs in labelled cells, with at least twice as many intracellular MNPs as there were extracellular MNPs across all 20 cells imaged (10 magplate- and 10 spin-labelled) (Figure 2G). These data indicate that our magplate labelling protocol enabled the efficient intracellular uptake of MNP labels.

Altogether, these data demonstrate that the use of our custom designed magplate results in efficient intracellular labelling of otherwise hard-to-label immune cell lineages with MNP labels. We therefore continued to use the magplate protocol for all cell labelling procedures in subsequent experiments.

### Magplate labelling CD8 T cells with MNPs does not adversely affect *in vivo* and *in vitro* cell function

**Figure 3:**
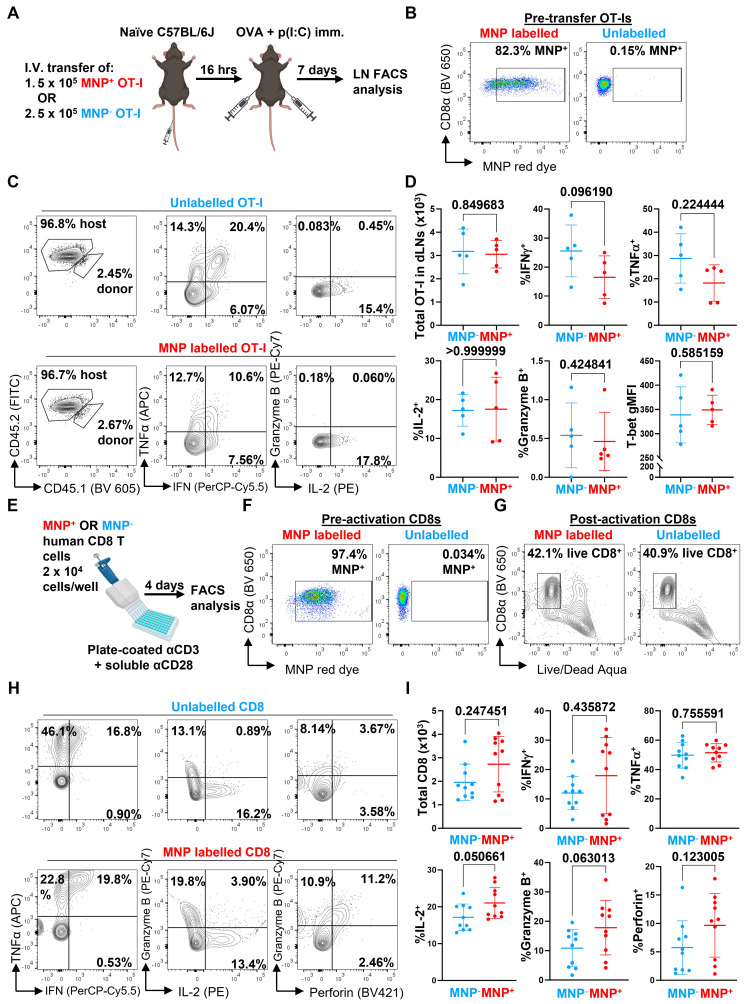
** MNP labelling does not significantly affect CD8 T cell function. (A)** OT-I CD8 T cells were labelled with MNPs for 3 h with the magplate (red) or left unlabelled (blue). 5 x 10^5^ MNP^+^ or MNP^-^ OT-I cells were then adoptively transferred into C57BL/6J mice by intravenous injection and the mice were immunised with OVA + p(I:C) the following day by subcutaneous injection into both flanks. After 7 days, cells suspensions prepared from draining inguinal lymph nodes (LNs) were analysed by flow cytometry following restimulation with 5 μg/mL OVA SIINFEKL peptide + protein transport inhibitors. **(B)** Pre-transfer flow cytometry analysis of labelling efficiency in MNP^+^ (red) and MNP^-^ (unlabelled, blue) OT-I CD8 T cells. Gated on live CD8α^+^ events. **(C and D)** Representative flow cytometry plots (C) and quantification (D) of parameters of OT-I CD8 T cell effector function in inguinal LNs of mice. Gated on live TCRβ^+^ CD8α^+^ CD4^-^ events. Data are means ± SD and are from one experiment with five mice per group. **(E)** CD8 T cells isolated from healthy donor PBMCs were labelled with MNPs for 3 h with the magplate (red) or left unlabelled (blue). CD8 T cells were then activated *in vitro* using plate-coated αCD3 + soluble αCD28 antibodies and analysed by flow cytometry after 4 days. **(F)** Pre-activation flow cytometry analysis of labelling efficiency in MNP^+^ (red) and MNP^-^ (unlabelled, blue) human CD8 T cells. Gated on live CD8α^+^ events. **(G-I)** Representative flow cytometry plots (G and H) and quantification (I) of indicated readouts of CD8 T cell effector function following restimulation with PMA+ionomycin on day 4. Gated on total events **(G)** and live CD3^+^ CD8α^+^ CD4^-^ events **(H and I)**. Data are means ± SD and are pooled from two experiments with cells from different donors, with 4 and 6 replicates per group, respectively. Numbers indicate *p*-values of comparisons using two-tailed Mann-Whitney test.

We next evaluated whether the presence of the MNP label would affect the function of the labelled immune cells. We first transferred MNP-labelled and -unlabelled OVA-specific OT-I CD8 T cells separately into naïve C57BL/6J mice, then challenged the mice with subcutaneous injections of OVA + p(I:C) to locally activate the OT-I cells *in vivo* within the inguinal lymph nodes (LNs) draining the immunisation site (Figure 3A and B). After 7 days, we collected the inguinal LNs and analysed the infiltrating OT-I cells by flow cytometry for expression of CD8 T cell functional markers, including total OT-I cell numbers in the LNs, secretion of effector cytokines (IFNγ, TNFα, IL-2), expression of the cytotoxic molecule granzyme B, and expression of T-bet, a key transcription factor regulating CD8 T cell activation and differentiation (Figure 3C). We generally did not observe any significant differences in any of the measured parameters between *in vivo* activated MNP-labelled and unlabelled cells (Figure 3D); while there was a trend towards decreased secretion of IFNγ in MNP-labelled cells, this difference was not significant.

As a further test to determine whether MNP labelling adversely affected CD8 T cell function, we repeated our OVA+p(I:C) immunisation experiments in mice that received a 1:1 mix of MNP-labelled and -unlabelled OT-I CD8 T cells sourced from CD45.1^-^ CD45.2^+^ and CD45.1^+^ CD45.2^+^ mice, respectively (Figure S3A and S3B). At 6 days post-immunisation, we analysed the proportions of CD45.1^-^ CD45.2^+^ (MNP^+^) and CD45.1^+^ CD45.2^+^ (MNP^-^) OT-I cells within the CD8 T cell compartment of the inguinal LNs by flow cytometry. In this direct competition scenario, we observed a higher proportion of MNP^-^ compared to MNP^+^ OT-I cells (about 3.5 to 14.1 times higher) (Figure S3C and S3D, upper right). We also observed that T-bet expression was slightly but significantly reduced in MNP^+^ OT-I cells relative to MNP^-^ OT-I cells (Figure S3D, top right). However, secretion of effector cytokines (IFNγ, TNFα, IL-2), and expression of granzyme B and the activation marker CD44 were not significantly different between MNP^-^ and MNP^+^ cells (Figure S3C and S3D, middle and bottom rows), consistent with our previous observations (Figure 3C and 3D).

We also investigated whether labelling human CD8 T cells with MNPs affected their effector function. We isolated total CD8 T cells from healthy donor PBMCs, divided them into parallel MNP-labelled and -unlabelled groups, and activated them with αCD3 + αCD28 antibodies *in vitro* (Figure 3E and F). After 4 days, we analysed the activated cells by flow cytometry using a panel of CD8 T cell functional markers, including total CD8 T cells recovered, secretion of effector cytokines (IFNγ, TNFα, IL-2), and expression of the cytotoxic molecules granzyme B and perforin (Figure 3G and H). Similar to our previous results with mouse CD8 T cells, we did not observe any significant differences in any of the measured parameters (Figure 3I).

### Increased MNP labelling of cells using the magplate is mediated by magnetophoretic forces acting on MNPs

**Figure 4:**
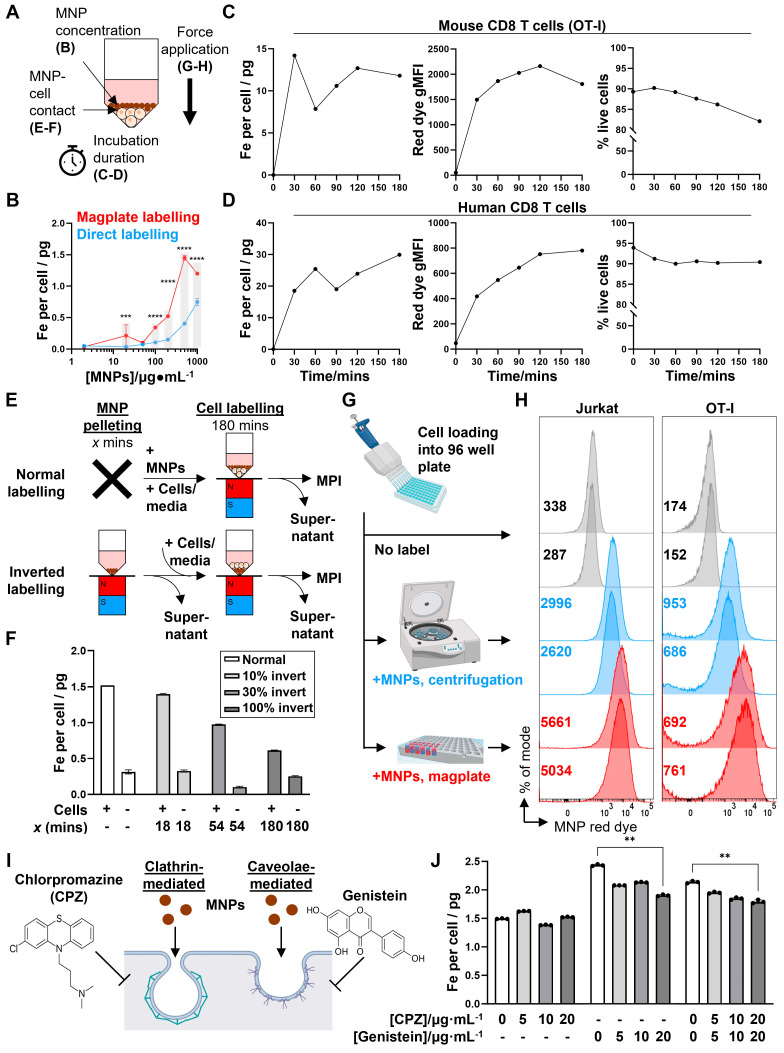
** Evaluation of potential mechanisms of magplate MNP labelling. (A)** Potential physical factors affecting MNP labelling of cells and relevant figure panels. **(B)** Iron label uptake measured by MPI in Jurkat cells that were labelled with MNPs at indicated concentrations for 3 h with (red) or without (blue) using the magplate. Data are means ± SD of three replicates, analysed using 2-way ANOVA with Sidak's test for multiple comparisons. **p < 0.01, ***p < 0.001, ****p < 0.0001. **(C and D)** Label uptake (MPI and flow cytometry red dye gMFI measurements) and viability (DAPI exclusion) of **(C)** primary mouse CD8 T cells (OT-I) and **(D)** human CD8 T cells after magplate labelling with 200 μg·mL^-1^ MNPs for indicated durations. MPI data are means of three repeat measurements from one sample each with ≥3 × 10^5^ live cells. Flow cytometry measurements are from one sample each with ≥5 × 10^4^ MNPs for indicated durations. **(E)** Inverted labelling procedure to assess whether proximity of MNPs and cells was sufficient for efficient label uptake. MNPs were concentrated at the bottom of wells of a 96-well V-bottom plate for indicated durations using the magplate. After removing the supernatant, Jurkat cells were layered over the MNPs and co-incubated for a further 3 h. **(F)** Iron uptake (MPI) in Jurkat cells following inverted and normal labelling with MNPs. Data are means ± SD of three repeat measurements from one sample each. **(G and H)** Jurkat (left) and primary mouse CD8 T cells (OT-I, right) were labelled as indicated in **(G)** and label uptake was assessed by flow cytometry measurements of the MNP red dye gMFI (numbers indicated on histograms). Data are from duplicate wells in one experiment.** (I and J)** Jurkat cells were labelled with MNPs with the magplate in the presence of increasing doses of chlorpromazine and/or genistein (inhibitors of clathrin- and caveolae-mediate endocytosis, respectively) and iron label uptake was measured by MPI. Data are means ± SD of three repeat measurements from one sample each, analysed using two-tailed Kruskal-Wallis test with Dunn's test for multiple comparisons relative to the no inhibitor control condition. **p < 0.01.

We then investigated potential physical and biological mechanisms that mediated the increased efficiency of MNP labelling using the magplate protocol. We hypothesised that one or several of four interrelated physical mechanisms could account for increased labelling efficiency: (1) increased MNP concentrations, (2) increased duration of incubation of cells with MNPs, (3) contact proximity between MNPs and cells, and (4) the application of force on the MNPs in the direction of the cells (Figure 4A). In our first experiments, we found that the mass of iron taken up by Jurkat cells with magplate labelling was almost always higher than that taken up by cells labelled by direct incubation, even at high MNP concentrations expected to be saturating (Figure 4B), with the amount of iron taken up per cell generally increasing with higher MNP concentrations used during labelling. This indicated that increasing MNP concentrations alone did not account for the increased efficiency of magplate labelling. We next tested whether increasing the duration of magplate application to increase accumulation of MNPs at the bottom of the plate (Figure 1C) was sufficient to increase MNP label uptake in primary mouse and human CD8 T cells.

We observed that label uptake (in terms of iron mass per cell and MNP red dye gMFI) increased with increasing labelling duration to plateau between 2 to 3 h across both cell types (Figure 4C and D, left and centre panels), with a small decline in cell viability as labelling duration was increased (Figure 4C and D, right panels). These suggest that a longer magplate incubation accumulated MNPs right next to the cell layer, thereby contributing to MNP label uptake.

We thus hypothesised further that the increase in contact surface area between MNPs and cells resulting from the increased accumulation of MNPs by the magplate over time was sufficient to increase the labelling efficiency. To test this, we first accumulated MNPs at the bottom of the plate wells with increasing durations of magplate labelling, then layered the cells over the accumulated MNPs for equal durations before measuring label uptake in the cells. This setup preserved the duration and surface area of contact between the cells and MNPs (Figure 4E). Contrary to our initial hypothesis, increasing the accumulation of MNPs on the well bottom before layering of cells on top (while preserving the duration of contact between MNPs and cells) did not result in increased MNP label uptake but instead decreased labelling efficiency (Figure 4F). This indicated that contact between cells and the larger MNP layer generated by the magplate was insufficient to increase labelling efficiency.

Therefore, we investigated the possibility that the magnetophoretic forces generated by the magplate on MNPs, acting in the direction of the cells, resulted in the active movement of MNPs into the cells as opposed to passive label uptake. To test this hypothesis, we devised an alternate method of applying a force on the MNPs acting in the direction of the cells by using centrifugation to generate a centrifugal acceleration of 800 x *g* on the MNPs (Figure 4G). We found that the application of either centrifugal or magnetic force resulted in increased MNP uptake as measured by flow cytometry (Figure 4H), across both the Jurkat T cell line and mouse primary CD8 T cells. In addition to physical mechanisms of MNP labelling, we investigated the potential contribution of endocytosis mechanisms to MNP label uptake. We used the small molecule inhibitors chlorpromazine (CPZ) and genistein to inhibit clathrin- and caveolae-mediated endocytosis, respectively, during 3 h magplate labelling of Jurkat cells (Figure 4I). Inhibition of caveolae-mediated endocytosis with genistein significantly decreased MNP uptake at the highest inhibitor dose (20 μg/mL), whereas inhibition of clathrin-mediated endocytosis with CPZ did not significantly decrease MNP uptake and had no additional effect when combined with genistein versus genistein incubation alone (Figure 4J). These results suggest that endocytosis uptake mechanisms likely contributed only a small fraction of total MNP label uptake during magplate labelling. Overall, our results indicate that the most likely dominant mechanism of MNP label uptake into cells during magplate labelling was the magnetophoretic force from the magplate acting on the MNPs towards the cells.

### Design and technical evaluation of a custom-built MPI device with handheld probe for whole body imaging of live mice

**Figure 5:**
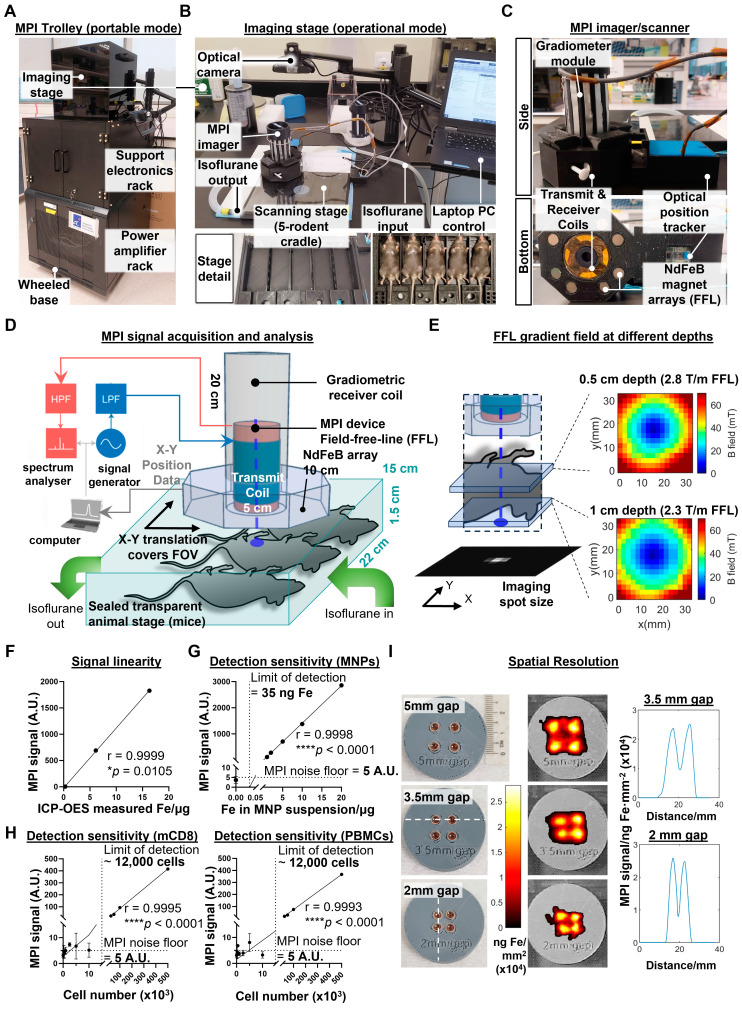
** A compact, portable magnetic particle imaging device for live bioimaging of mice. (A)** Magnetic Particle Imaging device built in four modular layers (top to bottom): an imaging stage (in portable mode), supporting electronics, power amplifiers, and a wheeled trolley base. **(B)** Imaging stage in operational mode, consisting of (top image, anti-clockwise beginning from top left): a camera for acquisition of optical images of specimens, a handheld single-sided MPI imager, a scanning stage with slots for up to five mice with isoflurane supply and exhaust tubes, and a laptop PC terminal to control the MPI scanning process. (Bottom images) Close-up detail of scanning stage before (left) and after (right) loading anaesthetised mice for imaging **(C)** Side and bottom close-up views of the handheld single-sided MPI imager showing the NdFeB permanent magnet arrays mounted inside that generate the vertically-oriented field-free-line (FFL) gradient, gradiometric transmit/receive coil assemblies, and the optical tracker for real-time tracking of the [X,Y] position coordinates of the FFL. **(D)** Illustration of signal inputs, outputs, and integration during the scanning process as the MPI imager is moved across the scanning stage within the optical field-of-view (FOV) containing the mice for imaging. **(E)** Mapping and characterisation of the field-free-line gradient strength measured by a 3-axis Teslameter (Lakeshore, USA) at indicated z-slice depths within the FOV volume space. **(F)** Pearson correlation of MPI signal versus gold standard ICP-OES quantification of iron mass **(G and H)** MPI measurements of titration series of MNP alone, MNP-labelled murine primary immune cells into unlabelled cells (5 x 10^5^ cells total) and MNP-labelled PBMCs to determine imaging sensitivity and limit of detection, analysed for Pearson correlation. **(I)** Characterisation of imaging spatial resolution using 4-well phantoms with progressively narrower gaps between the wells. The dashed lines in the photos mark the linear traces of the 1-dimensional line plots of MPI signal on the right.

The second challenge in developing MPI imaging for *in vivo* imaging of labelled immune cells was to construct an imaging device with the required signal linearity, detection sensitivity, and spatial resolution, while keeping it sufficiently small to be man portable. Our design solution fits all the power and signal electronics into a wheeled trolley base (Figure 5A), with the topmost stage used for imaging procedures (Figure 5B, top panel). The scanning stage maintains isoflurane anaesthesia for up to five mice simultaneously (Figure 5B, bottom panels), similar to the commonly used *in vivo* optical systems such as the IVIS (Perkin Elmer). A camera obtains the background photo to overlay with the MPI scans. Unlike commercial MPI scanners, our mechanically-shiftable single-sided scanner design can cover a FOV spanning the entire 5-mouse animal bed (200 x 100 x 15 mm), a marked improvement in FOV size over prior MPI scanners for rodents (40mm radius) 18.

To reduce the cost and complexity of the MPI imager/scanner, our design does not use the electromagnetic gradient shifting common in existing MPI systems 21. This eliminates the need for expensive power supplies to maintain and quickly move strong gradient fields across the FOV volume. Instead, our design maintains a field-free line (FFL) via a NdFeB permanent magnet array mounted concentric to the MPI transmit/receiver (Figure 5C). To raster the FFL across the FOV, the entire FFL array and transceiver is translated together across the X-Y plane, with the X-Y co-ordinates tracked in real time by an optical position tracker (Figure 5D). The FFL gradient strength is 2.3 T/m at 1cm depth (Figure 5E), comparable to the 2.35 T/m reported in prior projection MPI systems 38.

To validate the linearity of the MPI probe signal, we performed MPI measurements of three different cell samples (unlabelled, directly labelled, and magplate labelled) before quantifying total iron content in the samples by ICP-OES. MPI signal readings correlated positively with total iron measured by inductively coupled plasma-optical emission spectroscopy (ICP-OES) (r = 0.9999, Figure 5F). We then determined the detection sensitivity by serial dilution of MNPs in suspension, obtaining a limit of detection of 35 ng of iron (Figure 5G). We next determined the cellular limit of detection by measuring sample tubes with decreasing numbers of MNP-labelled cells titrated into unlabelled cells in suspension. For both mouse CD8 T cells and human PBMCs, the limit of detection that we obtained was approximately 12,000 labelled cells within a cell suspension of 500,000 total cells (2.4% of cell numbers) (Figure 5H).

Finally, we measured the spatial resolution of the MPI probe by using a series of 4-well phantoms with decreasing gap widths between the wells. Our MPI imager was able to resolve signal peaks spaced as closely as 2 mm apart (Figure 5I), comparable to previous MPI projection imagers using similar gradient strengths 19. Overall, our MPI imager design was able to achieve previous performance benchmarks for preclinical *in vivo* imaging without electromagnetically shifted gradients while increasing the scan throughput from 1 mouse to 5 mice per scan. While the trade-off is the inability to perform video-rate MPI imaging (it takes >10 min to scan across the entire FOV of 5 mice), this is acceptable for a system designed for longitudinal imaging studies with biological changes occurring over longer timescales.

### Longitudinal tracking of trafficking dynamics of antigen-specific CD8 T cell responses to immunisation using whole body MPI imaging

**Figure 6:**
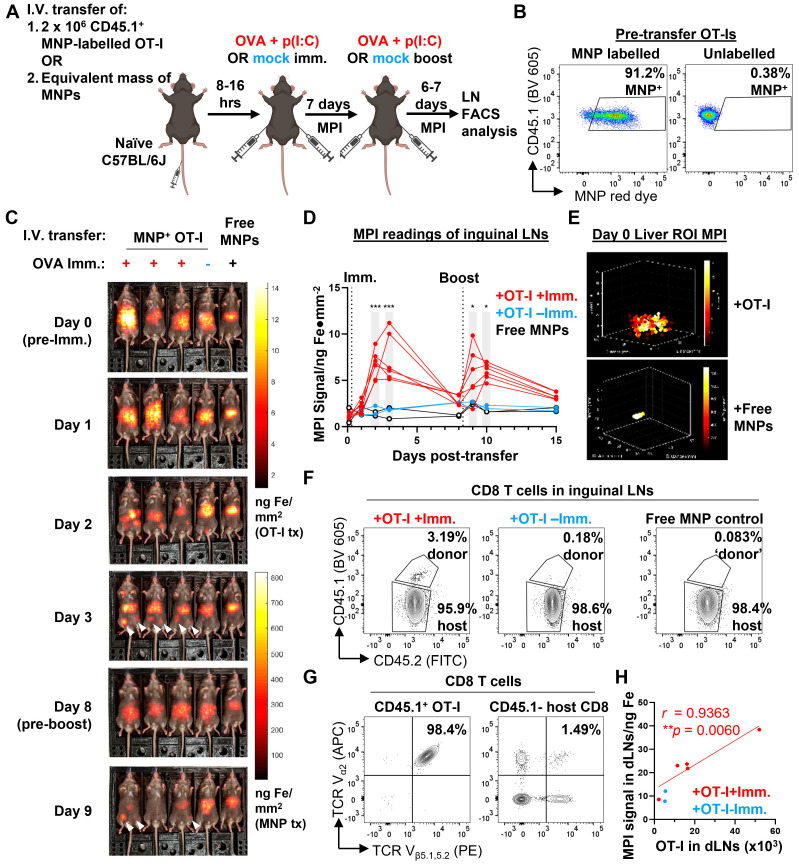
** MPI tracking of trafficking dynamics of adoptively-transferred antigen-specific CD8 T cells in response to immunisation. Imaging was conducted using the compact benchtop MPI device described in Figure 5. (A)** 2 x 10^6^ CD45.1^+^ CD45.2^+^ OT-I CD8 T cells were labelled with MNPs, then adoptively transferred into naïve C57BL/6J mice by intravenous injection. One mouse per set of five mice was injected with a suspension of an equivalent mass of free MNPs as a control. The following day, mice were immunised with subcutaneous injections of OVA + p(I:C) (immunisation, red) or PBS (mock immunisation, blue) into their flanks and similarly boosted after 7 days. Labelled cells were monitored by total body MPI throughout the entire duration with endpoint flow cytometry analysis of the draining inguinal lymph nodes (LNs). **(B)** Flow cytometry analysis of MNP labelling efficiency in OT-I CD8 T cells prior to adoptive transfer. Gated on total live cells. **(C)** Representative time course of MPI images of mice treated as in **(A)**. White arrowheads indicate local MPI signal peaks corresponding to the locations of the draining inguinal LNs. **(D)** Quantitative analysis of MPI measurements from regions of inguinal LNs of mice. Data are from one of two independent experiments, each with 6, 2, and 2 mice in the +OT-I+Imm. (red), +OT-I-Imm. (blue), and free MNP (black) groups, respectively. Statistical analysis using 2-way ANOVA with Sidak's test for multiple comparisons between the +OT-I +Imm. (red) and +OT-I-Imm. (blue) treatment groups at each time point, *p < 0.05, ***p < 0.001. **(E)** Representative 3D MPI images of abdominal regions of mice that received MNP^+^ OT-I CD8 T cells and free MNPs on day 0. **(F)** Representative flow cytometry plots for identification of transferred CD45.1^+^ OT-I CD8 T cells. Gated on live TCRβ^+^ CD8α^+^ CD4^-^ events. **(G)** Representative flow cytometry plots showing dominant co-expression of TCR V_α2_ and V_β5_ chains in OT-I cells but not in host CD8 T cells. Gated as shown in **(F)**. **(H)** Pearson correlation analysis of MPI signal in inguinal LN cell suspensions with total OT-I CD8 T cell numbers. Indicated numbers are for the +OT-I+Imm. group.

We next shifted to trials of live *in vivo* imaging of mice in model preclinical imaging scenarios (imaging was conducted using the compact benchtop MPI device described in Figure 5). First, we evaluated the robustness and usefulness of *in vivo* MPI imaging in tracking specific CD8 T cell responses to antigens. We designed an experiment in which adoptively transferred ovalbumin (OVA) peptide-specific OT-I CD8 T cells were induced to migrate to the inguinal lymph nodes by a local subcutaneous immunisation with OVA + polyinosinic:polycytidylic acid (p(I:C)) as an adjuvant (Figure 6A). After magplate labelling of CD45.1^+^ congenic OT-I cells and verification of labelling by flow cytometry (Figure 6B), labelled cells were transferred into healthy CD45.1^-^ OVA-naïve C57BL/6J wildtype mice and allowed to circulate *in vivo* for at least 8 h before immunisation with OVA + p(I:C) by subcutaneous injection into the ventral flanks. 7 days after the priming immunisation, mice received a booster immunisation with the same dose of OVA + p(I:C) and were monitored for a further 6-7 days before endpoint analyses of the inguinal lymph nodes (LNs) by flow cytometry.

Total body MPI imaging was performed throughout the entire two weeks of the immunisation + booster time course, with one mouse per cage of five mice left unimmunised as a control for the biodistribution of transferred OT-Is in the absence of OVA antigen challenge (Figure 6C, Figure S4A and S4B). In addition, to distinguish the biodistribution of MPI signals of MNP-labelled OT-I cells from that of any free MNPs present, we also intravenously injected one mouse per cage of five mice with an equivalent mass of iron in the form of free MNPs in suspension (Figure 6C, Figure S4A and S4B, rightmost mouse in image series).

We observed that MPI signal in the regions of interest (ROIs) of the inguinal LNs peaked between 1-3 days after the first immunisation dose before decreasing almost to baseline by 7 days after the immunisation dose in mice that received both MNP-labelled OT-Is and OVA + p(I:C) immunisation (Figure 6C and 6D and Figure S4A-C, day 1-3 time points). We did not observe any significant changes from baseline in MPI signal from inguinal LN ROIs in mice from either of the two control groups. As a further confirmation that the increase in MPI signal within the inguinal LN ROIs was due to an influx of labelled OVA peptide-specific OT-Is, we observed a second peak of MPI signal occurring after the booster antigen challenge than the first peak after the priming immunisation (Figure 6C and 6D and Figure S4A-C, day 9-10 time points). We did not observe similar changes in MPI signal from the inguinal LN ROIs of mice in either control group. We also detected bright MPI signal in the mid-to upper abdominal region of the mice. This signal was diffuse in mice that received labelled OT-I cells but localised to region corresponding to the anatomical region of the liver in mice that received free MNPs (Figure 6E and Figure S4D). As a secondary observation, the MPI signal from the liver ROI in mice receiving free MNPs decreased over time with kinetics that were best fit to a one-phase decay curve with a half-life of approximately 6.5 days (Figure S4E). These observations are consistent with the reported *in vivo* decay kinetics of free MNP labels cleared by the liver 39.

To verify that the MPI readings of the inguinal LN ROIs correlated linearly with the actual cell numbers of labelled OT-I cells in the LNs, we counted total transferred OT-I cells in cell suspensions prepared from whole inguinal LNs harvested on day 7. Transferred OT-I CD8 T cells were distinguished from endogenous CD8 T cells by expression of the congenic CD45.1 allele (Figure 6F). Expression of the OT-I transgenic TCR in the CD45.1^+^ CD8 population was confirmed by checking for co-expression of the TCR V_α2_ and V_β5_ chains, in contrast to CD45.1^-^ polyclonal host CD8 T cells, where only a small fraction of cells co-express the two TCR chains (Figure 6G). MPI signal of the total inguinal LN cell suspension correlated linearly with the total number of OT-I cells present (Pearson linear correlation r = 0.9363, Figure 6H), showing that MPI readings from the inguinal LNs were a quantitative indicator of the numbers of MNP-labelled cells present within the organ.

### Tracking adoptively transferred tumour-specific CD8 T cell responses in tumour-bearing hosts using whole body MPI imaging

**Figure 7:**
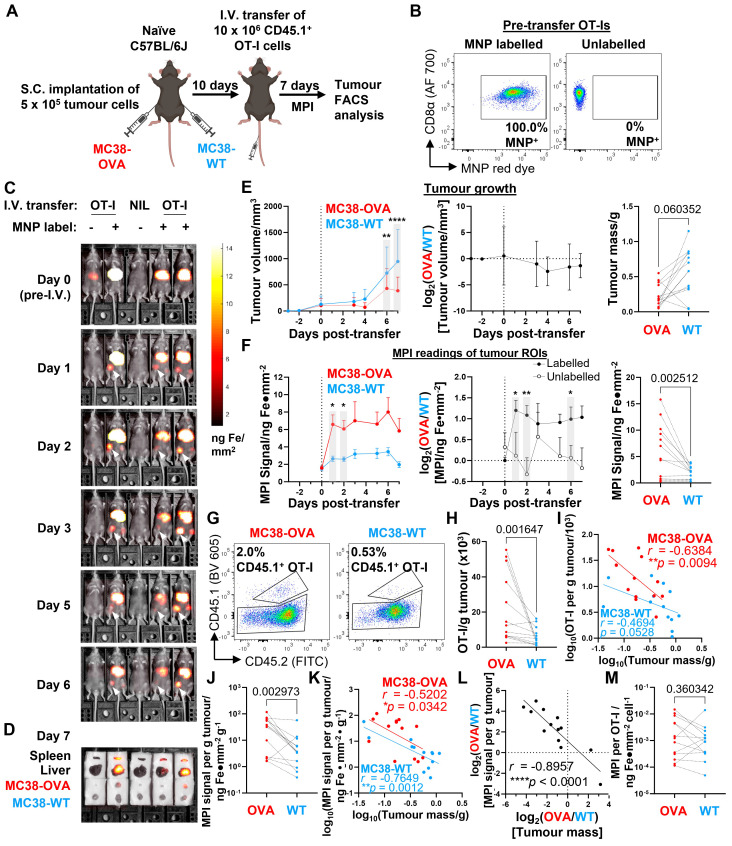
** MPI tracking of trafficking dynamics of tumour antigen-specific CD8 T cells transferred into tumour-bearing hosts. Imaging was conducted using the compact benchtop MPI device described in Figure 5. (A)** C57BL/6J mice were subcutaneously injected with 5 x 10^5^ OVA^+^ (red) and WT (blue) MC38 tumour cells into their right and left flanks, respectively. After 10 days, mice with dual palpable tumours received 10 x 10^6^ MNP-labelled CD45.1^+^ OT-I CD8 T cells by intravenous injection. Control mice received unlabelled OT-I CD8 T cells or PBS. Mice were imaged by total body MPI and tumours were analysed by flow cytometry at the endpoint. **(B)** Flow cytometry analysis of MNP labelling efficiency in OT-I cells pre-transfer. Gated on total live CD8α^+^ cells. **(C)** Representative MPI time course of mice treated as in **(A)**. White arrowheads indicate local MPI signal peaks corresponding to anatomical locations of MC38-OVA tumours. **(D)** *Ex vivo* MPI imaging of freshly dissected organs from the same mice in **(C)**, same colour scale as **(C)**. **(E and F)** Tumour growth **(E)** and MPI measurements **(F)** of mice that received MNP-labelled cells (*n* = 13). **(E)** (Left) Means ± SD of tumour volumes and (centre) relative (OVA/WT) tumour volumes. (Right) Endpoint tumour masses. **(F)** (Left) Means ± SD of MPI signal from tumour ROIs and (centre) relative (OVA/WT) MPI signal from mice receiving labelled or unlabelled cells. (Right) Endpoint tumour MPI measurements. **(G and H)** Representative flow cytometry plots **(G)** and quantification **(H)** of transferred CD45.1^+^ OT-I CD8 T cells in tumours. Gated on live TCRβ^+^ CD8α^+^ CD4^-^ events. **(I)** Correlation of infiltrating OT-I cell density against tumour masses. **(J)** Endpoint tumour MPI signal per unit mass. **(K)** Correlation of MPI signal per unit mass against tumour mass. **(L)** Correlation of relative (OVA/WT) MPI signal against relative (OVA/WT) tumour mass.** (M)** MPI signal per OT-I cell in tumours. Statistical analysis using 2-way ANOVA with Sidak's test for multiple comparisons (*p < 0.05, **p < 0.01, ****p < 0.0001) **(E and F** left, **F** centre), or using ratio paired t-test with the Benjamini-Krieger-Yekutieli FDR approach for multiple comparisons (*q*-values indicated)** (E and F** right, **H**, **J**, **M**). *r* and *p*-values indicated for Pearson correlation analyses **(I, K, L)**.

To complete our evaluation of the usefulness of MPI imaging in tracking adoptively transferred immune cells, we used MPI to track the infiltration of adoptively transferred tumour antigen-specific CD8 T cells in a preclinical tumour model that was permissive to T cell infiltration (imaging was conducted using the compact benchtop MPI device described in Figure 5). We first subcutaneously implanted OVA-expressing and wildtype MC38 murine adenocarcinoma cells (MC38-OVA and -WT, respectively) on opposite flanks of OVA-naïve C57BL/6J mice. After tumours became palpable, we adoptively transferred 10 x 10^6^ MNP-labelled CD45.1^+^ OVA peptide-specific OT-I CD8 T cells, monitored their *in vivo* trafficking by whole body MPI, and performed endpoint analysis of the cells by flow cytometry 7 days after OT-I cell transfer (Figure 7A). In addition to MNP-labelled OT-Is, we also injected a smaller number of mice with unlabelled OT-Is (Figure 7B). We then monitored the MPI signal intensities in MC38-OVA and MC38-WT tumour ROIs of the mice over 7 days (Figure 7C and Figure S5A), followed by *ex vivo* MPI imaging of freshly harvested liver, spleen, and MC38 tumours at the endpoint prior to organ dissociation (Figure 7D and Figure S5A).

We could detect MPI signal in the MC38-OVA ROIs of most mice that received MNP-labelled OT-Is (11 of 13) as early one day following adoptive cell transfer, whereas we only observed MPI signal in the MC38-WT ROIs of two of the same mice at that time point (Figure 7C and Figure S5A). MPI signal in the MC38-OVA ROI was always more intense than that of the contralateral MC38-WT ROI in the same mouse across all mice that received labelled OT-I cells. We continued to observe these findings throughout the time course of tumour growth (Figure 7E and 7F, left panels).

As expected, the transfer of OVA peptide-specific OT-I cells resulted in slower outgrowth of MC38-OVA tumours relative to MC38-WT tumours but did not completely eliminate the MC38-OVA tumours within 7 days (Figure 7E). In the control group of mice that received equal numbers of unlabelled OT-I cells, the outgrowth of MC38-OVA tumours was also slower than that of MC38-WT tumours on the same mice (Figure S5B), but there was no noticeable increase in MPI signal in their tumour ROIs (Figure S5C), nor was there an increase in the (OVA/WT) ratio of MPI signals from the MC38-OVA and -WT tumour ROIs, in contrast to the experimental group receiving MNP-labelled OT-Is (Figure 7F, centre panel). Unlike the previous immunisation experiments, liver ROI MPI signal decayed more rapidly with a best-fit half-life of 3.6 days (Figure S5D). At the end of the experiment, we observed that that the *ex vivo* MPI signal of harvested MC38-OVA tumours was overall significantly higher than that of paired MC38-WT tumours in the experimental cohort of mice receiving MNP-labelled OT-I cells (Figure 7F, right panel).

To confirm that the MPI signals detected in the tumours corresponded to the presence of MNP-labelled OT-I cells, we counted the total numbers of OT-I cells infiltrating all tumours by flow cytometry. Transferred OT-I cells were identified by CD45.1^+^ expression (Figure 7G) and checked for co-expression of TCR Vα_2_ and V_β5_ chains (Figure S5E), similar to our gating strategy for the immunisation experiments (Figure 4F and 4G) described in the previous section. As expected, MC38-OVA tumours had higher numbers of infiltrating OT-I cells per unit mass compared to their paired MC38-WT counterparts (Figure 7H), with higher densities of infiltrating OT-I cells correlating negatively with endpoint tumour mass in MC38-OVA tumours and MC38-WT tumours to a lesser extent (Figure 7I). We also observed that OT-I cells infiltrating MC38-OVA tumours expressed higher levels of PD-1 and TIM3 than OT-I cells infiltrating MC38-WT tumours in the same mouse as identified by expression of PD-1 and TIM3 (Figure S5F-H), consistent with antigen-specific activation within the OVA-expressing tumours.

Similar to our findings with infiltrating OT-I number densities, we also observed that the *ex vivo* MPI signal per unit mass of MC38-OVA tumours was significantly higher than that of MC38-WT tumours in most mice (12 of 13) (Figure 7J), and that the mass-normalised MPI signal also correlated negatively with tumour mass at endpoint (Figure 7K). Of note, we found a significant negative linear correlation between the (OVA/WT) ratios of MPI signals and tumour masses for each MC38-OVA and -WT tumour pair from the same mouse (r = -0.8957, Fig. 7L). In addition, we found that MPI signal per OT-I cell was overall not significantly different between paired MC38-OVA and -WT tumours, although there was considerable variation in ratios within the group (Figure 7M). To visualise the intratumoral distribution of MPI signals, we conducted a quantitative 3D MPI analysis of the MNP distribution at higher resolution within excised OVA+ and OVA- tumours harvested 7 days after transfer of labelled cells (imaging done immediately post-sacrifice on Day 7, Figure S6A). Our analysis revealed that MNPs were present within the tumour core (region defined as the middle 50% of the tumour radius, Figure S6B) for all 8 mice with significant MPI signal density >80% of the average signal density across entire tumour (including periphery), and that the MPI signal was not restricted to the periphery of the tumours (Figure S6C and D). Furthermore, there was no significant correlation trend between MPI signal and tumour mass (Fig. S6E). Taken together, our results suggest that MPI imaging measurements have utility in tracking the localisation of labelled cells *in vivo* and can be accurate reporters of clinically significant outcomes when used to track relevant populations of cell therapy products.

## Discussion and Conclusions

In our study, we developed two approaches to specifically address two major obstacles to the wider adoption of MPI for longitudinal imaging studies of transferred cells. First, we achieved a high efficiency in label uptake (~10 pg iron per cell) of MNPs into T cells within a mere 2-3 h using our magplate labelling protocol, which in turn enabled longitudinal *in vivo* MPI imaging for up to 2 weeks. Further optimisation showed that only 30 min was needed for an uptake of at least 8 pg iron per cell for both mouse and human T cells with magplate labelling. This was a significant improvement in labelling efficiency compared to recent MPI imaging studies on adoptively transferred immune cells, where 24 h incubation of label with the cells resulted in label uptakes of 1 and 3-5 pg of iron per cell, respectively 32,40. The labelling efficiency of 8 pg per cell achieved with magplate labelling within 30 min also surpassed the level of labelling achieved in a more recent study that used a rapid microfluidic method to mechanoporate T cells with MNPs (1 pg per cell within 10 min) 31,41. While our modification of MNPs by conjugating CPPs on the nanoparticle surfaces likely contributed to improved label uptake, we observed a 3-fold increase in label uptake with the magplate over direct incubation without the magplate for murine naïve CD8 T cells, validating that the magplate is a significant factor in the increased labelling efficiency.

A further important difference in the two protocols is that the microfluidic labelling method was optimised to use the clinically-approved MRI contrast Ferumoxytol, whereas we optimised our magplate protocol to use research-grade MPI-tailored MNPs such as Synomag™ nanoparticles, which give significantly better imaging performance (13-fold better sensitivity and 6-fold better resolution) in MPI compared to Ferumoxytol 42 and is a major candidate for contrast-agent approval as a MPI-tailored clinical tracer 43 when MPI systems complete their clinical translation. The primary mechanism of rapid label uptake into immune cells during magplate labelling was most likely the generation of a magnetophoretic force on the MNPs directed towards the cells by the magplate magnetic field. We were also able to increase MNP label uptake by using centrifugation as an alternate means to apply force on the MNPs (similar to the approach of a previous study 37), albeit at lower efficiencies and labelling rates. While it is not impossible to perform the labelling process of immune-cells-of-interest *in vivo*, reported efficiencies have been low compared to *in vitro* processes 44.

Second, we have developed a relatively simple, portable MPI imager that is 10 times smaller than existing commercial MPI scanners (preclinical MPI scanners from Bruker-Biospin, Ettlingen, Germany 45, and from Magnetic Insight Alameda, CA 46). This was accomplished by using permanent NdFeB magnet arrays instead of electromagnets to establish the magnetic field gradients required for MPI, and by designing a new scan trajectory where a single-sided MPI scanner is translated in the horizontal X-Y plane by the operator instead of using an in-bore scanner, thus significantly reducing power consumption without complex electronics and high-power electromagnets. The single-sided scanner design enabled a dramatic increase in the “lateral” field-of-view size, accommodating a 5-animal imaging bed to enable high-throughput scanning of mice. This is similar to the imaging setup in the commonly used IVIS® systems (Perkin Elmer) familiar to preclinical researchers.

This reduction in scanner complexity and footprint came with three trade-offs. First, the low-power, single-sided scanner design limits the scanning depth because of the natural drop-off of magnetic drive field strength with distance. However, the drive field strength was sufficient to permit accurate scanning up to 2 cm in depth, which is sufficient for typical mouse imaging studies. Second, the manually translated MPI scanner with a fixed permanent magnet array could not achieve the same rapid scan rates through the imaging FOV that commercial scanners using electromagnets can attain (about 46 frames per second). However, high scan speed and video rate visualisation are less critical for longitudinal imaging time courses spanning days or weeks. Third, the permanent magnets in our scanner could only generate a magnetic field gradient strength of 2.3 T/m, which is lower than the highest reported strength of 6.3 T/m generated using electromagnets 47 but is comparable to that generated by commercially available Bruker preclinical scanners (2.5 T/m maximal selection field strength). With this field gradient strength, our device could still resolve a gap of 2 mm between dots in an imaging phantom, which was sufficient for our *in vivo* experiments tracking immune cell localisation in mice as the smallest structures we imaged were lymph nodes with a typical diameter of 3-4 mm 48,49.

For sensitivity, we achieved a detection limit of about 12,000 mouse CD8 T cells because of the 10-fold increase in label uptake with our magplate labelling protocol (10 pg per cell). This resulted in an overall improvement in detection sensitivity compared to a 2021 MPI study with mouse CD8 T cells that reported a detection limit of 50,000 CD8 T cells with label uptake of 1 pg per cell 32. Further order-of-magnitude improvement of sensitivity and resolution may be achieved using newly-discovered superferromagnetic iron oxide nanoparticles with a reported >10-fold improvement in sensitivity and spatial resolution over existing optimal MPI nanoparticles 50,51.

Importantly, there was no significant effect on the viability or cell-intrinsic immune functionality of both mouse and human CD8 T cells despite the significantly higher uptake of MNP label. This is consistent with previous studies showing that the uptake of MNPs did not affect primary rat 52 and mouse T cells 53, and did not affect CAR-T cells generated from human PBMCs 31. In a head-to-head comparison, the cell-intrinsic effector phenotypes of MNP^+^ and MNP^-^ mouse CD8 T cells following *in vivo* activation were generally similar, although MNP^+^ CD8 T cells did not accumulate in LNs to the same degree that unlabelled CD8 T cells did in response to exposure to their cognate antigen. Interestingly, we only observed such differences in LN accumulation within a competitive setting (Figure S3), but not when MNP-labelled or -unlabelled cells were transferred into separate recipients (Figure 3A-D). One potential reason for this difference could be the fact that for the *in vivo* competition experiments, MNP^+^ and MNP^-^ CD8 T cells had to be sourced from different CD45-congenic mice to enable simultaneous tracking of two donor cell populations following adoptive transfer into host animals (Figure S3), in contrast to the preceding experiment where recipient mice received only MNP^+^ or MNP^-^ CD8 T cells sourced from the same donor (Figure 3A-D). Hence, the differences in LN accumulation in the *in vivo* competition experiment could have arisen from slight differences between the individual OT-I donor mice, despite our best efforts to control for these by age- and sex-matching the OT-I donor mice. Understanding the detailed biological reasons for these differences and refining our methods for cell MNP labelling and cell handling to mitigate these differences will be a major focus of future work.

We next validated our new MPI imaging platform by tracking the active migration of adoptively transferred OVA-specific mouse OT-I CD8 T cells to the LNs draining the site of a localised immunisation with OVA. Over the two weeks of each experiment, we observed the ingress of labelled OT-Is to the LNs after two successive doses of OVA+p(I:C) spaced about a week apart and found a quantitative linear correlation of MPI signal and total OT-I cell counts. We then further tested the limits of our MPI platform in a preclinical setting of adoptively transferred labelled OT-I cells as ACT for mice bearing dual OVA^+^ and OVA^-^ tumours. We observed a sustained higher MPI signal in the ROI of OVA^+^ tumours relative to OVA^-^ tumours in the same mouse, consistent with higher densities of infiltrating OT-I cells in OVA^+^ tumours relative to OVA^-^ tumours. Importantly, we found that the (OVA^+^/OVA^-^) ratio of MPI signal per unit mass of tumours was strongly negatively correlated with the (OVA^+^/OVA^-^) ratio of tumour masses.

Together, these longitudinal *in vivo* MPI studies highlight the potential for MPI-based metrics as real-time, non-invasive diagnostic biomarkers to evaluate the functional performance of ACTs. In our current study, we were unable to optimise conditions and identify detection reagents sensitive enough to detect the MNPs in tissue-infiltrating labelled T cells for histopathological analysis. Optimising protocols for the detection of MNP-labelled immune cells using histopathological analyses will be crucial for the discovery and validation of MPI-based biomarkers for diagnostic and prognostic assessment of cell therapy products *in vivo* and will be a key focus area of our future work. A second key direction of future will be detailed quantitative *in vivo* work using highly purified MNP-labelled cell populations to characterise the kinetics of *in vivo* trafficking in greater detail.

In conclusion, we anticipate that our robust MPI imaging workflow and platform will enable more thorough and sophisticated preclinical *in vivo* MPI studies using immune cells as ACTs for treating a larger diversity of solid tumour types than we have presented here. Our work also provides a practical starting point for the translational development of MPI systems as theranostic tools in the clinic for the monitoring and evaluation of new ACT therapies.

## Methods

### Mice

All mice were maintained in specific pathogen-free (SPF) conditions and used in accordance with guidelines of the A*STAR Biological Resource Centre Institute Institutional Animal Care and Use Committee (BRC IACUC). C57BL/6 were bred in-house by the SIgN Mouse Core Facility. CD45-congenic C57BL/6 mice and OT-I TCR transgenic mice were bred in-house by the investigators. All mice used as hosts in experiments were male mice 6-9 weeks of age. For adoptive transfer experiments, 6-12-week-old mice of both sexes were used as T cell donors.

### Cell culture and cell lines

Cell culture media and supplements were purchased from Gibco (Thermo Fisher Scientific). All primary cell cultures and Jurkat cells were cultured in RPMI 1640 media (RPMI) supplemented with 10% (v/v) foetal bovine serum (FBS), 2 mM glutamine, 100 U penicillin-streptomycin, 1 mM sodium pyruvate, 10 mM HEPES, and 50 μM 2-Mercaptoethanol. MC38 and its derived cell lines were grown in DMEM media supplemented with 10% (v/v) FBS, 1x GlutaMAX™, 100 U penicillin-streptomycin, and 10 mM HEPES. Jurkat and MC38 cell lines (the latter is referred to as MC38-WT in-text) were purchased from ATCC. The OVA-expressing MC38 tumour cell line (MC38-OVA) was generated by co-transfection of MC38-WT cells with a plasmid coding for a Sleeping Beauty transposase (Addgene plasmid #34879 54) and a transposase shuttle plasmid (derived from Addgene plasmid #20281 55) bearing an N-terminally truncated variant of chicken ovalbumin that was sub-cloned from pcDNA3-deltaOVA (Addgene plasmid #64595 56) and a blasticidin resistance cassette. 24 h after transfection, cells were selected in culture media containing 10 μg/mL Blasticidin S (Thermo Fisher Scientific) for 7 days. MC38-OVA cells were validated by their ability to induce proliferation of OVA-specific OT-I T cells *in vitro*. OVA expression in MC38-OVA was maintained by propagating the cell line in media containing 10 μg/mL Blasticidin S.

### Flow cytometry

Where required, single-cell suspensions were restimulated as indicated with PMA and Ionomycin using eBioscience™ Cell Stimulation Cocktail (plus protein transport inhibitors) (Thermo Fisher Scientific), or with 5 μg/mL of peptide plus eBioscience™ Protein Transport Inhibitor Cocktail (Thermo Fisher Scientific) for 4 h prior to evaluation by flow cytometry. Unstimulated samples (treated only with Protein Transport Inhibitor Cocktail) were acquired in parallel as gating controls. Single-cell suspensions were first stained with an amine-reactive live/dead exclusion dye or DAPI (5 mins, room temperature (RT)), followed by Fc receptor blocking (10 mins, RT), and then stained for surface epitopes with appropriate fluorophore-conjugated antibodies (20 mins, 4°C). Where required, cells were fixed and permeabilised with reagents from the eBioscience™ FoxP3/Transcription Factor Staining Buffer Set (Thermo Fisher Scientific) in preparation for intracellular staining with appropriate fluorophore-conjugated antibodies (20 mins, 4°C). Supplementary table 1 lists details of the labelling reagents used for flow cytometry in this work. FACS buffer was prepared from PBS by addition of 2% FBS (v/v) and 1mM EDTA. Perm buffer was prepared by dilution 10x Perm concentrate (eBioscience™ FoxP3/Transcription Factor Staining Buffer Set, Thermo Fisher Scientific) to 1x in distilled water. All flow cytometry data were acquired using an LSRFortessa X-20 or FACSymphony A3 and analysed using FlowJo software (BD Biosciences).

### Construction of magplate

96 Neodymium Iron Boron (NdFeB) permanent magnets (Lifton Magnets, 6mm diameter x 12mm height cylinder, Grade N50, Ni-Cu-Ni coated, approximate surface gauss 7131 G) were arranged in an alternating north-south configuration with positions aligned to the well positions of a standard 96-well tissue culture plate. The entire 12 x 8 array of magnets were mounted into a fused deposition modelling (FDM) 3D-printed housing made of polylactic acid (PLA) material (3D Aura, 1.75mm PLA filament) with dimensions of 127.71 mm length x 85.43 mm width x 12 mm height. These dimensions are compliant with standards set by the ANSI-/SLAS to ensure a proper “stacking” fit and compatibility with most commercially-available 96-well plates for MNP labelling. The surfaces of the NdFeB magnets are positioned within 2mm of the well bottom of the tissue culture plate, and the resulting magnetic gradient strength along the vertical axis is on average 82 mT/mm within 5mm of the magplate surface.

### Chemical Modification and Preparation of MNP for Cell Labelling

70 nm diameter Synomag® nanoparticles containing redF fluorescent dye (excitation/emission wavelengths: 552/580 nm) and with -NH_2_ surface modification (synomag-CLD-redF-NH2, micromod Partikeltechnologie GmbH, Germany) were chemically conjugated with cell-penetrating peptides (CPPs) via sulfhydryl-reactive crosslinker chemistry with the cysteine group on the peptide. The synthetic CPP used was derived from amino acids 47-57 of the Human Immunodeficiency Virus Trans-activator of Transcription (TAT) protein (TAT_p47-57_) (amino acid sequence GRKKR-RQRRR-GYKC) (Axil Scientific, >95% purity by HPLC). First, MNPs first underwent buffer exchange with filter-sterilised borate buffer (50mM sodium borate pH 8.4, 5mM EDTA) using a PD-10 desalting column pre-packed with Sephadex G-25 gel filtration medium (Cytiva). Next, the linker molecule N-Succinimidyl Iodoacetate (SIA, Thermo Fisher Scientific) was dissolved in DMSO to a concentration of 42mg/mL and added to the purified MNPs at a mass ratio of 21 mg SIA: 10 mg MNP. The mixture was allowed to react in the dark at room temperature for 15 min then excess SIA was removed by Sephadex G-25 column purification. Finally, the synthetic CPP was dissolved in borate buffer and slowly added dropwise to the suspension of SIA-MNPs achieving a final stoichiometry of 8 mg TAT_p47-57_ per 10 mg MNP, then incubated overnight at 4^o^Cprotected from light. Next, excess TAT_p47-57_ was removed using Sephadex G-50 (Sigma-Aldrich) gel purification column and eluted into base RPMI 1640 medium (Gibco). The entire procedure was performed in a dark environment due to the light-sensitive nature of SIA and to minimise photobleaching of the red dye within the MNPs. Unless otherwise indicated, all steps were performed at room temperature.

### Isolation of primary immune T cells for MNP labelling

For isolation of mouse primary CD8 T cells (wildtype polyclonal and OT-I TCR transgenic), total cell suspensions were first prepared by mechanical dissociation of spleens and lymph nodes using 70 μm cell strainers with RPMI media. CD8 T cells were then magnetically isolated by negative selection using reagents from the EasySep™ Mouse CD8+ T Cell Isolation Kit (Stemcell Technologies) according to the manufacturer's instructions.

For isolation of human primary immune cells, de-identified human blood tissue was collected in accordance with and under the following project: HSA Residual Blood Samples for Research, project titled “Harnessing immune response for new therapies in transplantation and cancer” (Ref. No. 201306-04). PBMCs were isolated by Ficoll gradient centrifugation (400 x *g*, 30 mins with brake off) from healthy donors. Where required, CD8 T cells were then magnetically isolated by negative selection using reagents from the EasySep™ Human CD8+ T Cell Isolation Kit (Stemcell Technologies) according to the manufacturer's instructions.

### MNP labelling procedure

Cells for MNP labelling were resuspended in RPMI + 1% (v/v) FBS at 2-3 x 10^6^ cells/mL. 100 μL of cell suspensions (containing 2-3 x 10^5^ cells) was loaded into the wells of a sterile non-TC coated 96-well V-bottom plate (Wuxi NEST Biotechnology Co., Ltd). Cells were quickly brought to the bottom surfaces of the wells by quick centrifugation (~1000 rpm, ≤ 10 s) in a benchtop centrifuge. 100 μL of a 400 μg/mL MNP suspension (prepared as described in preceding sections) was then gently layered on top of the cells without disturbing the cell pellet and the plate with 200 μL total volume per well was quickly centrifuged again (~1000 rpm, ≤ 10 s). Where indicated, 100 μL of unsupplemented RPMI media was loaded onto wells containing cells to serve as MNP-unlabelled controls. For magplate-labelling, the fully loaded 96-well plate was then mounted on a magplate (magplate labelling) or left as is (direct labelling) and transferred to a 37°C cell culture incubator for 3 h (standard labelling duration) or other indicated durations. For spin-labelling by centrifugation, the fully loaded 96-well plate was centrifuged continuously in a benchtop centrifuge at 800 x *g* for 3 h at 32°C.

At the end of the labelling, cells were collected from the wells and the empty wells were washed once with 200 μL PBS per well, with the PBS wash pooled together with the original contents of the wells. The cells were then centrifuged at 800 x *g* for 5 min and the pellet was washed a second time with an excess of PBS with a second centrifugation of 800 x *g* for 5 min. Resulting cell pellets were then resuspended in appropriate media, counted, and prepared for further work as required. A small aliquot of unlabelled and labelled cells was analysed by flow cytometry to assess label uptake by fluorescence intensity measurements of the MNP red dye in the 561 nm laser-excited PE channel (585/15 nm bandpass filter).

### Live cell fluorescent imaging

Primary mouse OT-I CD8 T cells were first labelled with carboxyfluorescein succinimidyl ester (CFSE) prior to MNP labelling. Cells were resuspended at 2 x 10^7^ cells/mL in PBS at room temperature, and an equal volume of 2 mM CFSE in PBS was added into the middle of the cell suspension, mixed gently, and incubated at 37°C for 2 min. The labelling reaction was then quenched with a 5-fold excess volume of RPMI medium with 10% FBS (v/v). Cells were then recovered by centrifugation at 1500 rpm for 5 min and labelled with MNPs as described in the previous section. Following recovery after MNP labelling, cells were resuspended at 2 x 10^6^ cells/mL in RPMI with 10% FBS, and 100 μL of this cell suspension was loaded into wells of a clear, flat-bottomed 96 well plate. Image acquisition was performed using a EVOS FL Auto2 imaging system (Thermo Fisher) at 400x magnification. Post-acquisition image analysis was performed using Fiji (NIH).

### Transmission electron microscopy imaging

Primary mouse OT-I CD8 T cells were labelled with MNPs using either the magplate or spin labelling protocol. Cell pellets were collected and washed once with sterile PBS before overnight fixation in a solution of 4% Paraformaldehyde + 2.5% glutaraldehyde (Ted Pella, Inc.) in PBS, pH 7.3. Fixed cell pellets were then washed in buffer, post-fixed for 1 h in 1% osmium tetroxide + 1.5% Potassium Ferrocyanide in PBS, then dehydrated in an ethanol series, absolute acetone and embedded in Araldite 502 resin (Ted Pella, Inc). Ultra-sections were cut at 100nm on a Leica EM UC6 Ultramicrotome, collected on 200 mesh copper grids covered with a Formvar carbon support film (Electron Microscopy Sciences), and stained for 8 mins in lead citrate stain. Photographs were taken with Transmission Electron Microscopy (Tecnai G2 Spirit Biotwin, FEI company) at 100kV with a bottom mounted high-sensitivity FEI Eagle 4k digital camera. Post-acquisition image analysis was performed using Fiji (NIH).

### *In vitro* experiments to determine mechanism of magplate labelling

Jurkat cells were incubated with MNPs at indicated concentrations for 3 h with or without the magplate to determine the effect of varying MNP concentration on label uptake (Figure 4B). To determine the effect of increasing labelling duration on label uptake, primary mouse and human CD8 T cells were incubated with 200 μg/mL MNP labels for indicated durations (Figure 4C and 4D). To evaluate whether increasing the contact surface area between MNPs and cells increased the MNP label uptake, we pre-concentrated MNPs from a 200 μg/mL suspension on the bottom of 96-well V-bottom plates before layering cells on top (Figure 4E and 4F) and co-incubated the MNPs and cells for 3 h with the magplate. To investigate whether application of mechanical force was sufficient for label uptake into cells, we layered cells in 96-well V-bottom plates, then overlaid the cells with a 200 μg/mL MNP suspension per the standard labelling procedure as described in a previous section. Replicate plates were either incubated at 37°C on a magplate or centrifuged at 800 x *g* at 32°C in a benchtop centrifuge for 3 h. To ascertain the contribution of endocytosis to MNP label uptake, we incubated Jurkat cells in the presence of chlorpromazine (CPZ) and/or genistein at indicated concentrations for 3 h with a 200 μg/mL MNP suspension.

### High-throughput handheld MPI imaging scanner for mice

A custom vertical field-free line (FFL) 2.8T/m/μ0 magnetic particle imager was built and used for this study. The imager utilises a single-sided configuration adapted from a previously validated tabletop MPI design 57, and incorporates larger gradiometric multi-layer receiver coils (outer diameter: 30 mm) concentrically housed within drive coils (outer diameter: 50 mm). The MPI imager uses a drive field frequency of 0.25 kHz with an excitation strength of 28 mTpp that is almost the same as one of the optima (1 kHz 28mTpp) for MPI sensitivity, resolution and safety elucidated in an earlier MPI parametric optimisation study 57. As the frequency is lower than the cited human-safe optima, the MPI imager used in this study is operating at human-safe parameters. The FFL is created by an array of Neodymium Iron Boron alloy (NdFeB) permanent magnets (Lifton Magnets, 20mm cube, Grade N50, approximate surface gauss 6408 G) and mechanically translated in the X-Y plane to raster the FFL across the entire field-of-view volume. To provide a flat X-Y surface for smooth rastering of the imager FFL in the X-Y plane, a thin, optically transparent, and rigid acrylic plate (thickness: 1 mm) was placed between the MPI imager and the 5-mouse animal bed of the imaging stage. Image reconstruction of a 2D maximal-intensity-projection image was performed by conventional X-space image reconstruction which assigns the MPI pixel intensity value to the instantaneous [X, Y] position of the MPI device, which was continuously tracked by a custom-modified high-performance optical mouse circuit board integrated into the imager device (Logitech, >10000 dpi, ~1kHz refresh rate). After a 2D image was obtained, the third dimension of depth was obtained by a harmonic analysis and 1D deconvolution process due to the drop-off of applied drive field strength (normalised to the average field strength of 28mTpp) with z-depth being correlated to a change in the harmonic ratios of the MPI signal. Finally, to output a background co-registered 2D maximal-intensity-projection image, the MPI pixel intensity in 2D was directly superimposed upon a camera photo image of the scan subjects in the 5-mouse animal bed. This camera image was acquired by a detachable web camera (Logitech, 1080p) connected to the PC laptop controller mounted on a snap-to swing arm. Accurate co-registration between the camera photo and MPI image was ensured by a 3-point alignment calibration between the photo position of fiducial markers at the corners of the animal bed and MPI image coordinates of the same markers.

### MPI *in vivo* imaging of mice and *ex vivo* organ imaging

All animal imaging procedures were approved by the A*STAR Biological Resource Centre Institute Institutional Animal Care and Use Committee (BRC IACUC) in line with AAALAC guidelines. To prepare for *in vivo* scanning, mice were first anaesthetised using a (2:1) mix of 1-3% isoflurane and air-oxygen at a flow rate of 1.5L/min in a dedicated anaesthesia induction chamber, then immediately transferred to the 5-mouse animal bed of the MPI device imaging stage. To keep the mice sedated during imaging, (0.5 - 1.5%) isoflurane was supplied to each mouse via the gas manifold and nose cones built into the imaging stage (Figure 5E, bottom right). The isoflurane was contained within the animal bed by covering the bed with a thin, optically transparent acrylic plate, which permitted the acquisition of optical images via the camera module and served as a window to monitor the breathing of the scanned mice. The rigid acrylic plate also functioned as a plane for the smooth X-Y translation of the MPI imager device as it rastered across the X-Y FOV. Following the acquisition of the optical image of the scan subjects in position, the MPI imaging scans were be performed by mechanically rastering the imager device across the field-of-view containing 5 mice lined up side-by-side (200 mm width x 100 mm length x 15 mm height). For *ex vivo* organ imaging, organs of interest were harvested and laid out on white surgical gauze covered with waterproof Parafilm™ to improve background visual contrast in the photo image, then scanned by the MPI imager as previously described. Quantitative 3D MPI imaging of excised tumours was conducted using the same imaging procedure in the previous subsection but zoomed-in to a narrow 3D field-of-view (FOV) around the excised tumours demarcated by the dashed red lines in the 2D photo background. The depth dimension was reconstructed by a harmonic analysis and 1D deconvolution process due to the drop-off of applied drive field strength (normalised to the average field strength of 28mTpp) with z-depth being correlated to a change in the harmonic ratios of the MPI signal. The data was then plotted using MATLAB software (Mathworks, Natick) in 3D using a spot size of 2mm corresponding to the gradient-defined spatial resolution limits of the MPI device. Quantitative analysis of the average signal density in the separate core versus shell ROI of the tumours was performed by (1) defining the core as the middle region of the tumour demarcated by a spherical surface with half the average radius of the whole tumour, (2) binning the signal voxels into their respective ROI, and finally (3) computing the average signal density for each core and shell ROI (“MPI signal”, in units of ng Fe·mm^-3^) as well as the signal density of core and shell ROIs normalised relative to the signal of the total tumour ROI (“Normalised MPI signal density”, dimensionless). The indicated statistical comparisons were then performed using GraphPad Prism software.

### Quantification of cellular MNP uptake by MPI *in vitro* measurement and inductively coupled plasma-optical emission spectroscopy (ICP-OES)

Cellular MNP uptake was measured *in vitro* by interrogating labelled cells with the MPI drive waveform at 0.25 kHz and 28 mTpp (same scan parameters as *in vivo* imaging). To quantify iron label uptake, MPI signals of standard samples containing between 0-200 μg of MNPs were measured to obtain a calibration curve. After subtracting background signal (average of MPI signal measurements of the 0 μg standard) across all measurements, a conversion factor (MPI signal per μg MNP) corresponding to the gradient coefficient of the best-fit line was obtained and used to calculate the MNP masses in experimental samples. The label uptake per cell was then calculated by dividing the total MNP mass by the number of cells (measured by counting using Trypan Blue exclusion). To quantify MNP iron content, cell pellet samples were prepared for ICP-OES by digestion in 1 mL of 5N hydrochloric acid to liberate iron ions from the MNPs, then diluted to a final volume of 20 mL in deionised water and acquired on a Avio200 ICP-OES machine (Perkin Elmer).

### OVA immunisation studies

One day before immunisation, OVA-specific OT-I CD8 T cells were isolated and labelled with MNPs as described earlier. Labelled or unlabelled OT-I cells were resuspended in PBS and transferred by intravenous injection into recipient OVA-naïve C57BL/6J mice as indicated. The following day, recipient mice were immunised by subcutaneous injection of 10 μg OVA (Imject™ Ovalbumin, Thermo Fisher Scientific) + 50 μg poly(I:C) (Sigma Aldrich) in 50 μL PBS into each flank. As controls, some mice were instead given 50 μL of PBS only into each flank as a mock immunisation. Where indicated, a booster dose with an equal mass of OVA + poly(I:C) in PBS or a mock boost of PBS only was given to each mouse in the experimental and control groups, respectively.

Total body MPI imaging of all mice was performed over the time course of the experiment, with particular focus on the time points from 0 to 2 days after each OVA immunisation. At the endpoint of the experiment, total cell suspensions were prepared from inguinal lymph nodes and re-stimulated with 5 μg/mL OVA SIINFEKL peptide (Sigma Aldrich) for 4 h prior to analysis by flow cytometry. Where indicated, single sample MPI measurements of lymph node cell suspensions were also taken.

### Tumour-specific CD8 T cell trafficking studies

MC38-WT and MC38-OVA tumours were implanted into the left and right flanks of healthy C57BL/6J, respectively, by subcutaneous injection of 5 x 10^5^ cells in PBS at each tumour site. After 10 days, when tumours were palpable, MNP-labelled or -unlabelled CD45.1^+^ OT-I cells were intravenously transferred into tumour-bearing mice for 7 days prior to endpoint analysis. Total body MPI imaging of all mice was performed over the time course of the experiment. At the endpoint of the experiment, *ex vivo* MPI imaging and weighing of freshly harvested spleens, livers, and tumours was performed. Total cell suspensions for flow cytometry analysis were then prepared from tumours by dissociation using a gentleMACS^TM^ Octo Dissociator with Heaters (Miltenyi Biotech) in C tubes with the '37C_m_TDK_1' programme in an enzyme mix containing 1 mg/mL collagenase D (Sigma Aldrich), 20 U/mL DNAse I (Sigma Aldrich), and 100 μg/mL hyaluronidase Type V (Sigma Aldrich).

## Supplementary Material

Supplementary figures and table.

## Abbreviations

DAPI: 4',6-diamidino-2-phenylindole, dilactate
gMFI: geometric mean of fluorescence intensity
irAE: immune-related adverse events
IVIS: *in vivo* imaging system
LN(s): lymph node(s)
Magplate: magnetic 96-well plate
MNP(s): magnetic nanoparticle(s)
MPI: magnetic particle imaging
MRI: magnetic resonance imaging
PET: positron emission tomography
FLI: fluorescence imaging
BLI: bioluminescence imaging
SPIO: superparamagnetic iron oxide
FFL: field-free-line
FFP: field-free-point
FOV: field of view (imaging)
NdFeB: neodymium-iron-boron alloy
OT-I: ovalbumin peptide-specific OT-I TCR transgenic
PBMC: peripheral blood mononuclear cells
OVA: ovalbumin
CPP: cell-penetrating-peptide
p(I:C): polyinosinic:polycytidylic acid
CPZ: chlorpromazine
ROI(s): region(s) of interest
TEM: transmission electron microscopy
ICP-OES: inductively coupled plasma - optical emission spectroscopy
TCR: T cell receptor
WT: wildtype
