# Supplementary material for: High-efficiency magnetophoretic labelling of adoptively-transferred T cells for longitudinal in vivo Magnetic Particle Imaging
Source: Theranostics. 2024 Sep 23;14(16):6138–60. doi: 10.7150/thno.95527 (PMC11488102; doi:10.7150/thno.95527)
Supplement: Supplementary file 1 — Supplementary figures and table. [file thnov14p6138s1.pdf]

Supplementary Material

High-efficiency magnetophoretic labelling of adoptively-transferred T cells for longitudinal in vivo Magnetic Particle Imaging

Rong En Tay[1*†], Lokamitra P[1,2], Shun Toll Pang[2^], Kay En Low[3], Hui Chien Tay[1], Charmaine Min Ho[1], Benoit Malleret[3,4], Olaf Rötzschke[1,4,5], Malini Olivo[6] & Zhi Wei Tay[2,7*†‡]

[1]Singapore Immunology Network (SIgN), Agency for Science, Technology and Research (A*STAR), 8A Biomedical Grove, #04-06 Immunos, Singapore 138648, Republic of Singapore.

[2]Institute of Bioengineering and Bioimaging (IBB), Agency for Science, Technology and Research (A*STAR), 11 Biopolis Way, #02-02 Helios Building, Singapore 138667, Republic of Singapore.

[3]Electron Microscopy Unit, Yong Loo Lin School of Medicine, National University of Singapore, Singapore 117597, Republic of Singapore.

[4]Department of Microbiology and Immunology, Immunology Translational Research Programme, Yong Loo Lin School of Medicine, National University of Singapore, Republic of Singapore.

[5]School of Biological Sciences, Nanyang Technological University, 50 Nanyang Avenue, Singapore 639798. Republic of Singapore.

[6]A*STAR Skin Research Labs (A*SRL), Agency for Science, Technology and Research (A*STAR), 31 Biopolis Way, #07-01 Nanos, Singapore 138669, Republic of Singapore.

[7]National Institute of Advanced Industrial Science and Technology (AIST), Health and Medical Research Institute (HMRI), 1-2-1 Namiki, Tsukuba, Ibaraki 305-8564, Japan.

*Correspondence: zhiwei.tay@aist.go.jp and tay_rong_en@immunol.a-star.edu.sg

[†]These authors contributed equally

Current affiliations: ^Institute of Technical Education, 2 Ang Mo Kio Drive, Singapore 567720, Republic of Singapore

‡Health and Medical Research Institute (HMRI), National Institute of Advanced Industrial Science and Technology (AIST), 1-2-1 Namiki, Tsukuba, Ibaraki 305-8564, Japan

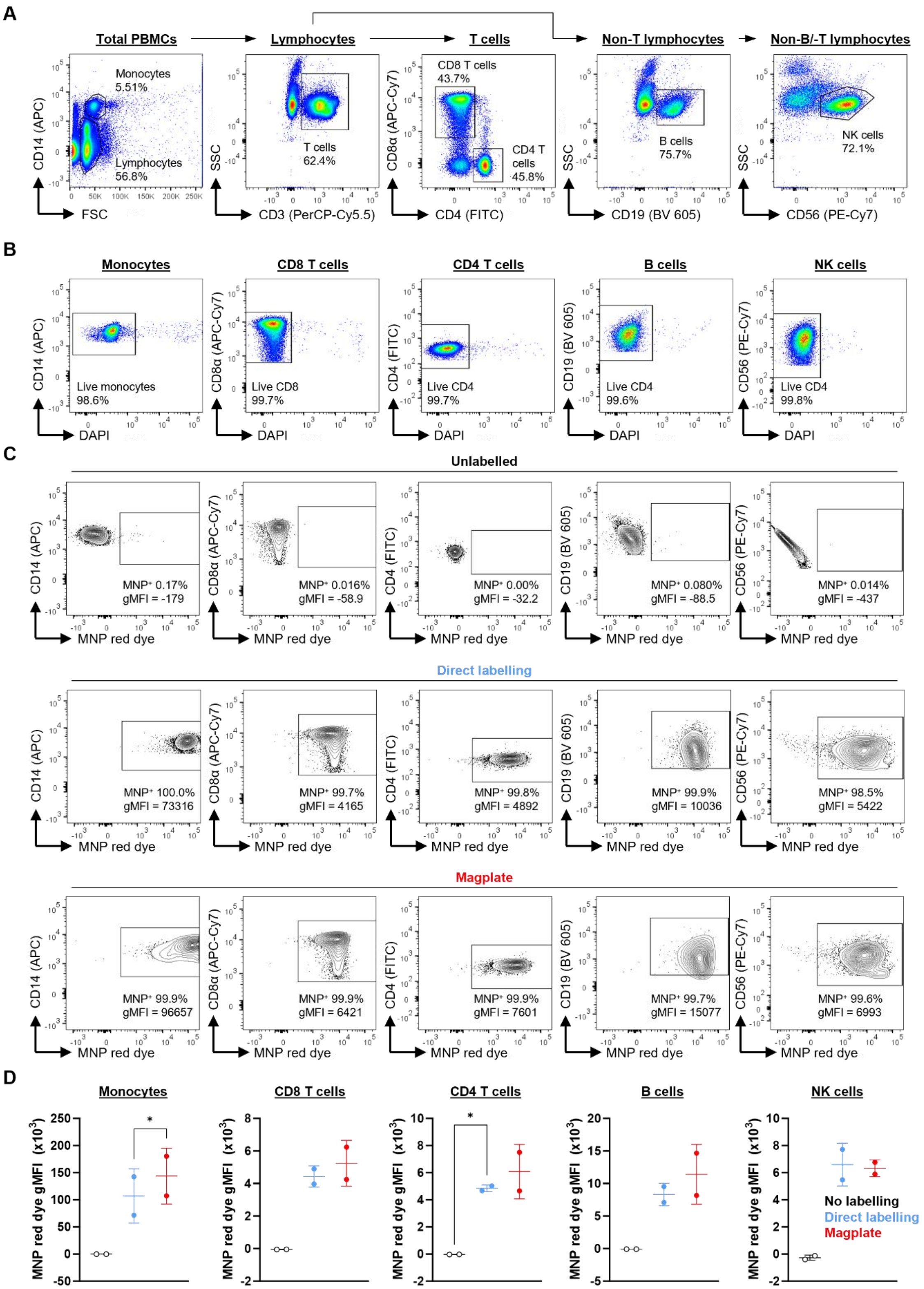

Figure S1. Magplate labelling increases MNP label uptake across five major subsets of healthy human PBMCs, related to Figure 1. (A-D) PBMCs from two healthy human donors were labelled with MNPs either by direct labelling or magplate labelling for 3 h. As a control, parallel samples of PBMCs were incubated for 3 h without MNP labels. Label uptake within five major subsets of immune cells in the

PBMCs was then assessed by flow cytometry **(A)**. Representative flow cytometry plots showing gating strategy to identify (left to right) monocytes, CD8 and CD4 T cells, B cells, and NK cells in PBMCs. Data are representative of labelled and unlabelled PBMCs from two separate donors. **(B)** Live/Dead exclusion by DAPI staining for the five immune cell subsets, gated as in **(A)**. **(C)** Representative flow cytometry plots showing gating and MNP label uptake in the five immune cell subsets, gated as in **(A)**, in PBMC samples that were (top to bottom rows) left unlabelled, directly labelled, and magplate-labelled. **(D)** Quantification of label uptake across immune cell subsets from PBMCs from two healthy donors. Data are mean ± SD, statistical analysis using two-tailed one-way ANOVA with Tukey's test for multiple comparisons. *$p < 0.05$.

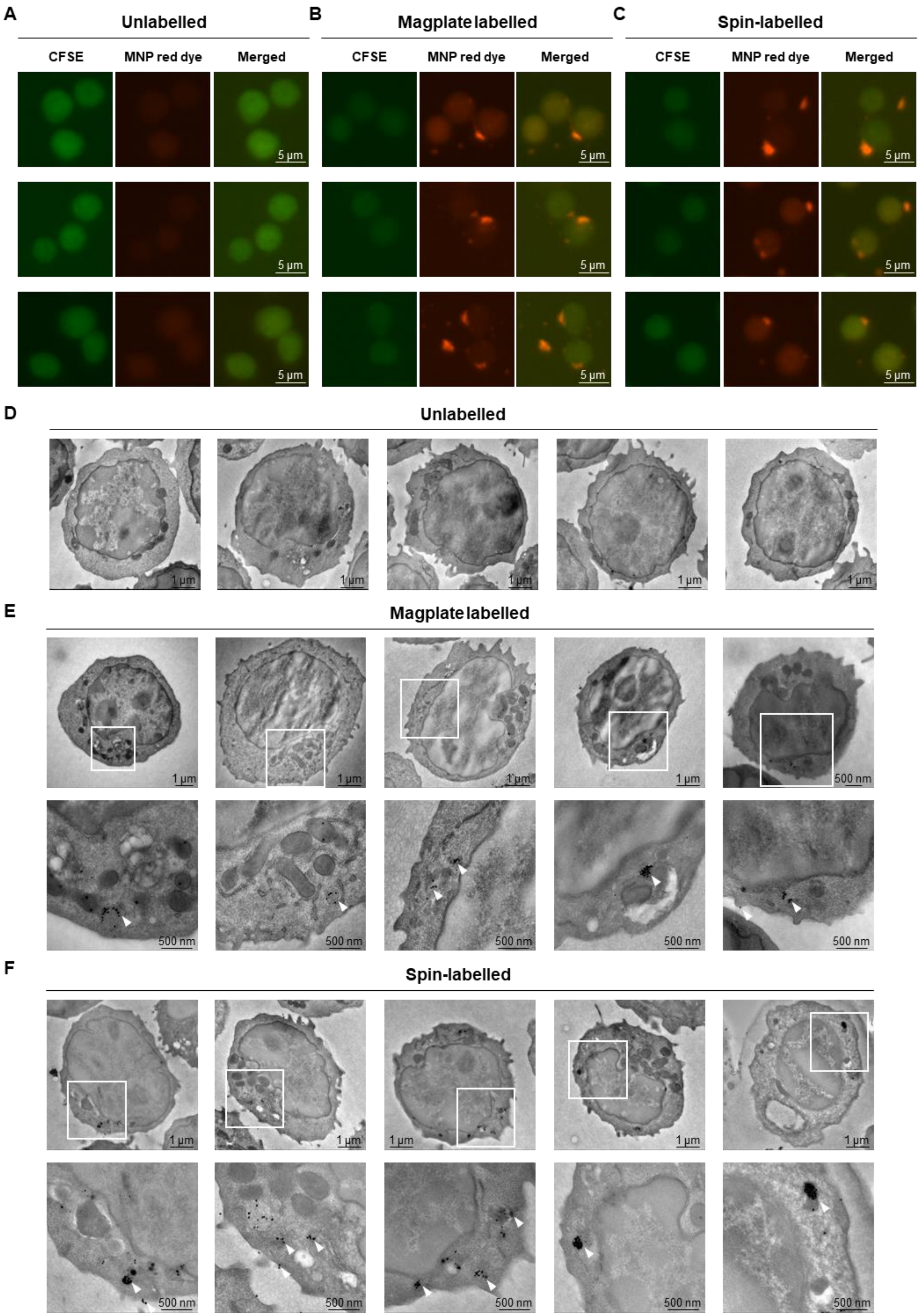
A
Unlabelled
B
Magplate labelled
C
Spin-labelled
CFSE
MNP red dye
Merged
5 μm
D
Unlabelled
1 μm
E
Magplate labelled
500 nm
F
Spin-labelled

46

Figure S2. Extended sets of live cell microscopy and transmission electron microscopy images of MNP-labelled primary mouse CD8 T cells, related to Figure 2 (A-C) Representative live cell fluorescent images of primary mouse OT-I CD8 T cells labelled with cytosolic CFSE (green channel) prior to indicated labelling procedures. **(D-F)** Representative transmission electron microscope images of primary mouse OT-I CD8 T cells treated with indicated labelling procedures. White squares indicate regions of interest displayed at higher magnification below each whole cell image **(E and F)**. White arrowheads indicate MNPs in high-magnification images (**E and F**).

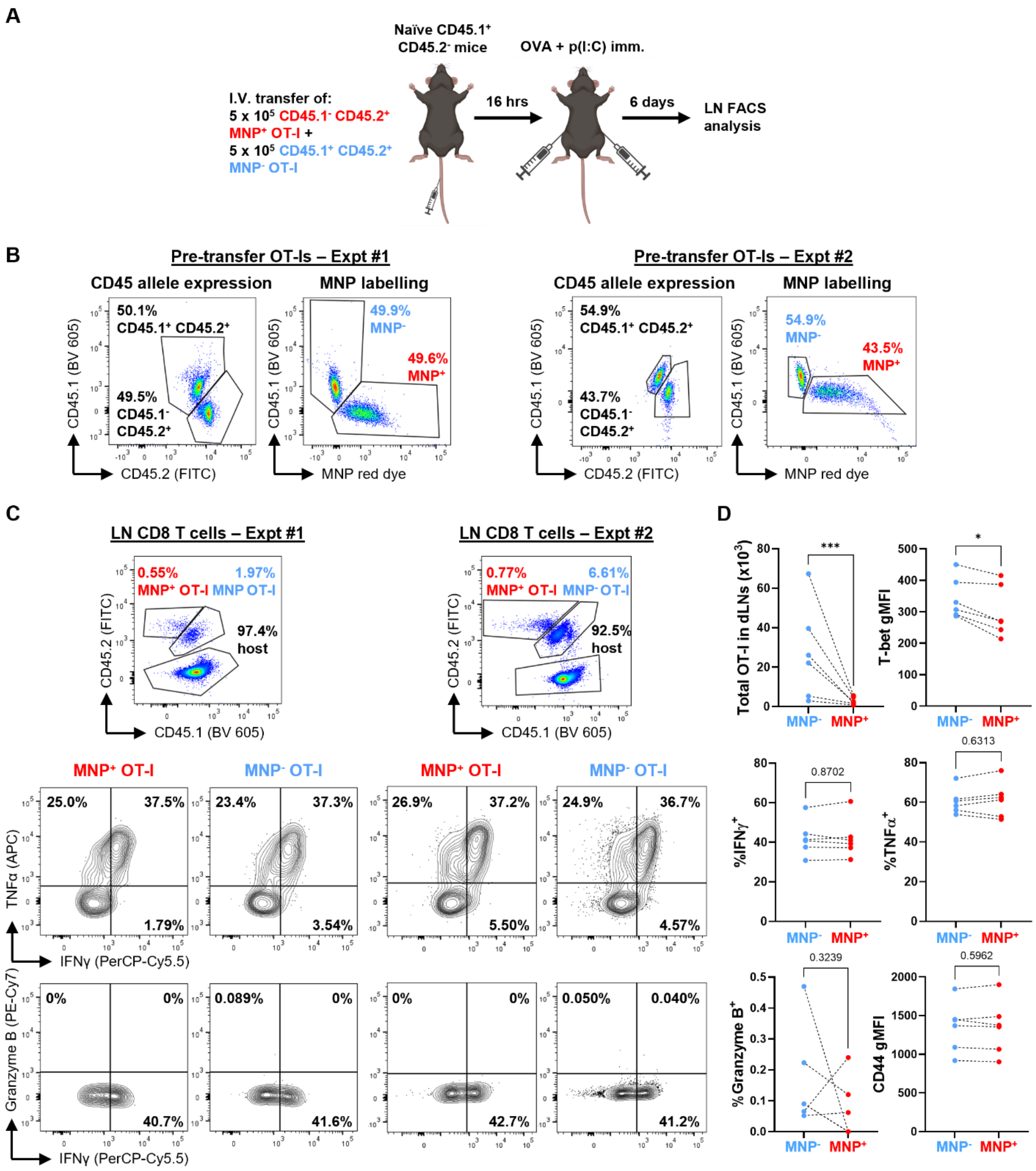

Figure S3. In vivo competition experiment between MNP-labelled and –unlabelled mouse primary CD8 T cells, related to Figure 3. (A) OT-I CD8 T cells harvested from CD45.1^{-} CD45.2^{+} mice were labelled with MNPs for 3 h with the magplate (red). In parallel, OT-I CD8 T cells harvested from CD45.1^{+} CD45.2^{+} mice were left unlabelled for 3 h (blue). 5 x 10^{5} MNP^{+} and MNP^{-} OT-I cells were then adoptively transferred into CD45.1^{+} CD45.2^{-} congenic C57BL/6J mice by intravenous injection and the mice were immunised with OVA + p(I:C) the following day by subcutaneous injection into both flanks. After 6 days, cells suspensions prepared from draining inguinal lymph nodes were analysed by flow cytometry following restimulation with 5 µg/mL OVA SIINFEKL peptide + protein transport inhibitors.

(B) Pre-transfer flow cytometry analysis of the 1:1 mix of CD45.1$^-$ CD45.2$^+$ MNP$^+$ OT-I (red) + CD45.1$^+$ CD45.2$^+$ MNP$^-$ OT-I (blue). Gated on live CD8α$^+$ events. **(C and D)** Representative flow cytometry plots (C) and quantification (D) of parameters of OT-I CD8 T cell effector function in inguinal lymph nodes of mice. Gated on live TCRβ$^+$ CD8α$^+$ CD4$^-$ events. Data are means ± SD and are from two independent experiments with two and four recipient immunised mice, respectively. Numbers indicate *p*-values of comparisons using two-tailed ratio paired t-test (all parameters except % Granzyme B$^+$) or two-tailed paired t-test (% Granzyme B$^+$).

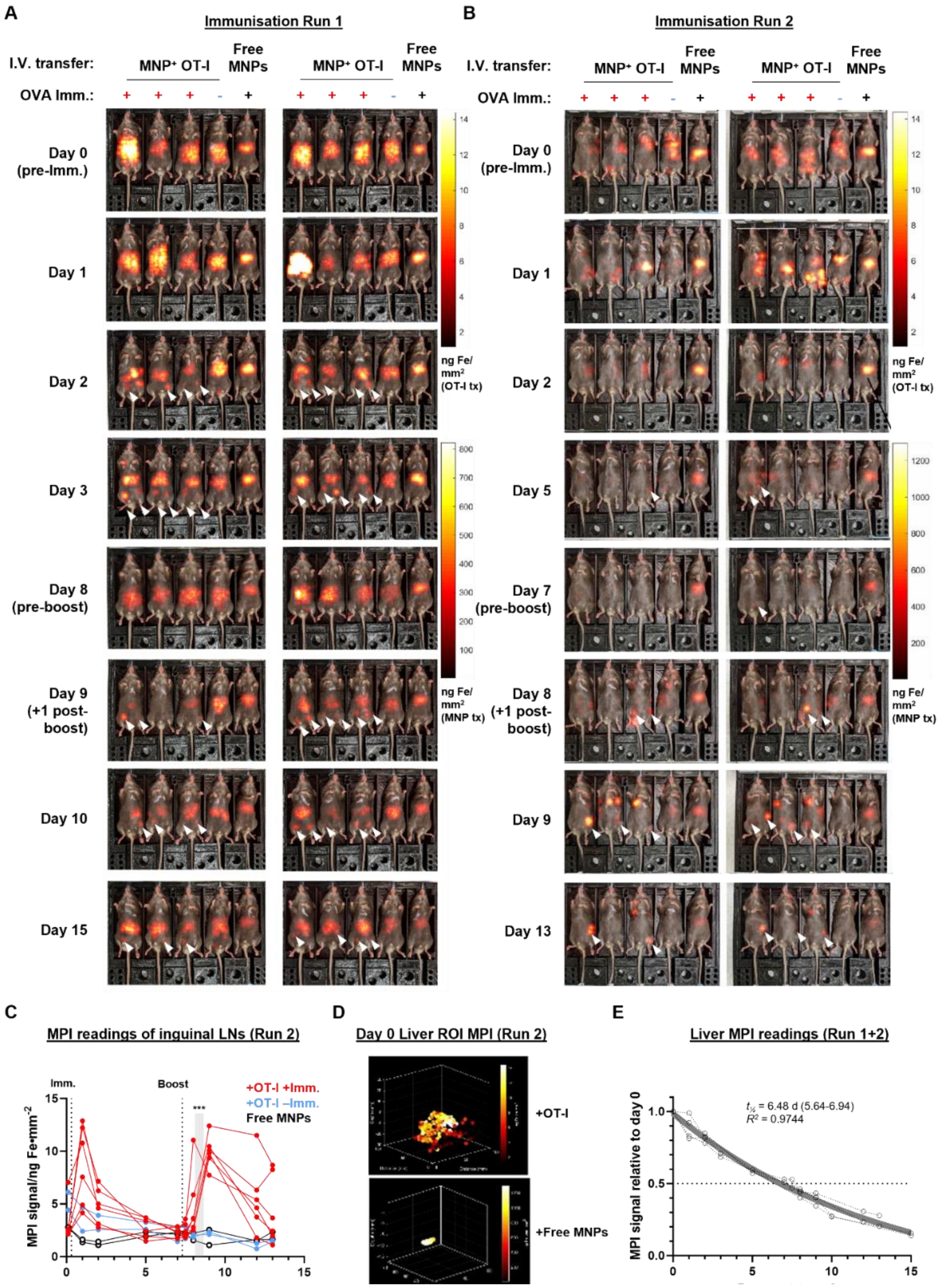
A
Immunisation Run 1
I.V. transfer:
MNP⁺ OT-I
Free MNPs
OVA Imm.:
Day 0 (pre-Imm.)
Day 1
Day 2
Day 3
Day 8 (pre-boost)
Day 9 (+1 post-boost)
Day 10
Day 15
ng Fe/mm² (OT-I tx)
ng Fe/mm² (MNP tx)
B
Immunisation Run 2
Day 5
Day 7 (pre-boost)
Day 8 (+1 post-boost)
Day 9
Day 13
C
MPI readings of inguinal LNs (Run 2)
Imm.
Boost
***
+OT-I +Imm.
+OT-I −Imm.
Free MNPs
MPI signal/ng Fe•mm⁻²
D
Day 0 Liver ROI MPI (Run 2)
+OT-I
+Free MNPs
E
Liver MPI readings (Run 1+2)
t½ = 6.48 d (5.64-6.94)
R² = 0.9744
MPI signal relative to day 0

72

Figure S4. Additional data from MPI tracking of adoptively-transferred antigen-specific CD8 T cells in response to immunisation, related to Figure 6. (A and B) MPI imaging time courses of two independent immunisation experiments with 10 mice each. 2 x 10^6 $CD45.1^+$ $CD45.2^+$ OT-I CD8 T cells were labelled with MNPs, then adoptively transferred into C57BL/6J mice ($CD45.1^-$ $CD45.2^+$) by intravenous injection. As an imaging control, one mouse in each set of five mice was injected with a suspension of free MNPs of an equivalent mass of iron. The following day, the mice received subcutaneous flank injections of OVA + p(I:C) (immunisation, red) or PBS (mock immunisation, blue) and were boosted 7 days later as shown in **Figure 6A**. Total body MPI was performed throughout the duration of the experiment. White arrowheads indicate local MPI signal peaks corresponding to the anatomical locations of the inguinal lymph nodes draining the immunisation sites. **(C)** Time course of MPI signal in inguinal lymph nodes of mice from **(B)**, with 6, 2, and 2 mice in the +OT-I+Imm. (red), +OT-I-Imm. (blue), and MNP (black) treatment groups, respectively. Statistical analysis by 2-way ANOVA with Sidak's test for multiple comparisons between the +OT-I +Imm. (red) and +OT-I-Imm. (blue) treatment groups as indicated by the grey bars. ***$p < 0.001$. **(D)** Representative 3D MPI images of abdominal cavities of mice from **(B)** that received OT-I CD8 T cells and free MNPs on day 0. **(E)** Decay of MPI signal relative to day 0 in liver ROIs of mice that received intravenous injections of free MNPs (two per experiment, four total). Grey curve shows the best-fit one-phase decay curve, with the best-fit and (95% confidence interval) of the half-life ($t_{½}$) and R^2 goodness-of-fit as indicated.

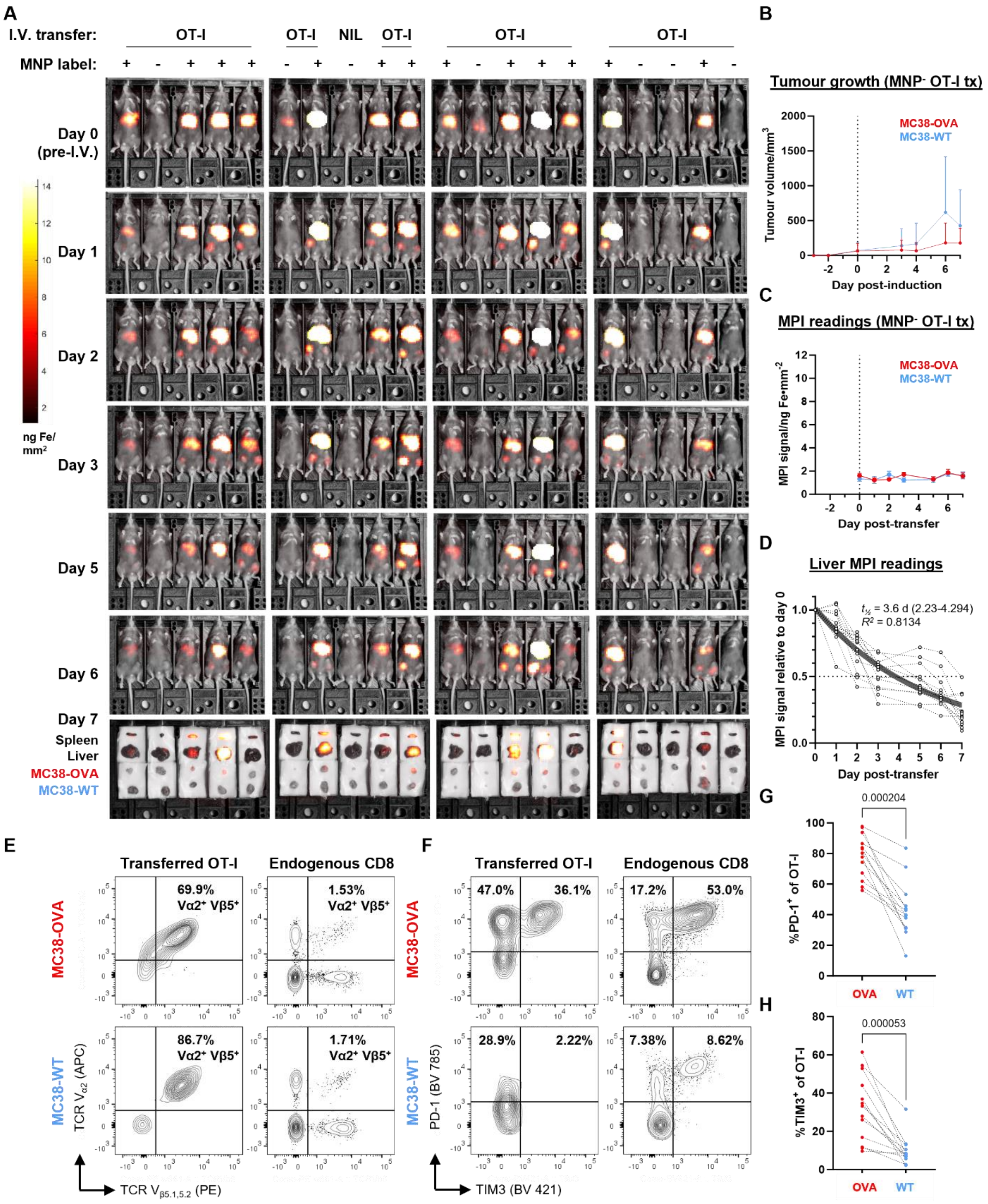

Figure S5. Additional data from MPI tracking of tumour antigen-specific CD8 T cells in tumour-bearing hosts, related to Figure 7. (A) MPI imaging time course of all mice in the experiment shown in **Figure 7.** C57BL/6J mice received subcutaneous implantation of 5 x 10^5 MC38-OVA and -WT tumour cells injected into their right and left flanks, respectively. After 10 days, mice with dual palpable tumours

received 10 x 10^6 MNP-labelled $CD45.1^+$ $CD45.2^+$ OT-I CD8 T cells by intravenous injection (*n* = 13). As controls, some mice received unlabelled OT-I CD8 T cells (*n* = 6), and one mouse was injected with PBS (mock cell transfer). Total body MPI was performed throughout the duration of the experiment and endpoint *ex vivo* MPI was performed on freshly harvested organs on day 7 post-cell transfer. **(B and C)** Tumour growth data **(B)** and MPI signal data **(C)** from mice that received unlabelled OT-I CD8 T cells (*n* = 6). Statistical analysis using (left) 2-way ANOVA with Sidak's test for multiple comparisons between MC38-OVA and -WT tumour volumes with matching for tumour pairs from the same mouse (no significant differences detected. **(D)** Decay of MPI signal relative to day 0 in liver ROIs of mice that received MNP-labelled OT-I CD8 T cells (*n* = 13). Grey curve shows the best-fit one-phase decay curve, with the best-fit and (95% confidence interval) of the half-life ($t_{½}$) and R^2 goodness-of-fit as indicated. **(E)** Representative flow cytometry plots showing positive identification of OT-I cells in tumours from mice that received MNP-labelled OT-I cells by co-expression of TCR $V_{\alpha 2}$ and $V_{\beta 5}$ chains. Gated on $TCR\beta^+$ $CD8\alpha^+$ $CD4^-$ CD45.1+ events as shown in **Figure 7G**. **(F-H)** Representative flow cytometry plots **(F)** and quantification **(G and H)** of T cell exhaustion marker expression in OT-I cells in tumours from mice that received MNP-labelled OT-I cells (*n* = 13). Gated on $TCR\beta^+$ $CD8\alpha^+$ $CD4^-$ CD45.1+ events as shown in **Figure 7G**. Statistical analysis using ratio paired t-test with the Benjamini-Krieger-Yekutieli FDR approach for multiple comparisons between paired MC38-OVA and -WT tumours from the same mouse (number indicates *q*-value)

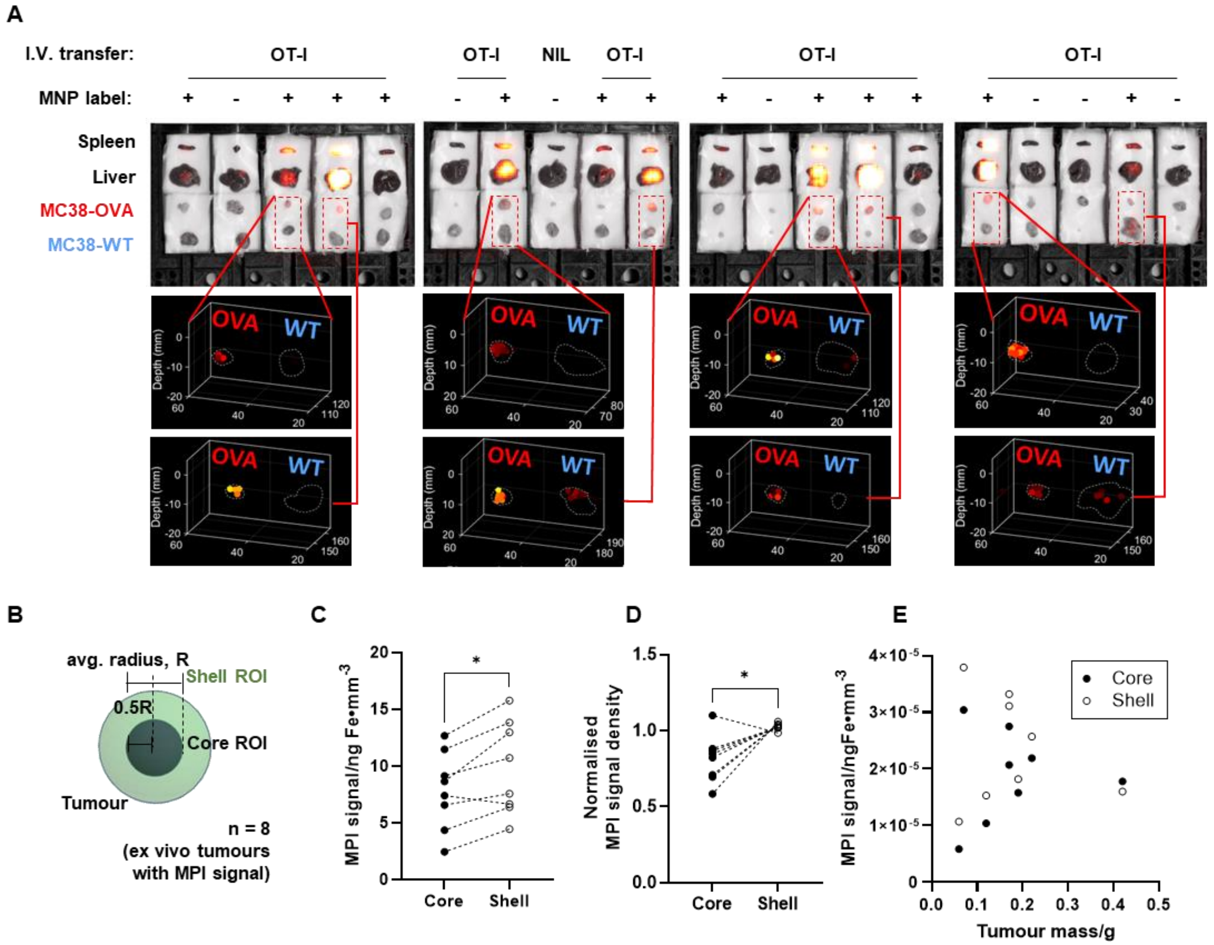

Figure S6. Quantitative 3D image analysis of MNP biodistribution within excised tumours, related to Figure 7. (A) MPI imaging in 3D of excised tumours from the same experiment presented in Fig. 7. Only mice with labelled OT-I CD8 T cells and the excised tumour having detectable MPI signal at Day 7 (n = 8) in the MC38-OVA tumour were selected for zoom-in imaging. A narrow field-of-view (FOV) around the excised tumours demarcated by the dashed red lines in the 2D photo background was then plotted in 3D with MC38-OVA and MC38-WT (OVA$^-$) tumours in the field-of-view with spot size set as 2 mm corresponding to the spatial resolution limit of 2 mm of the MPI device used. Tumour outlines within each FOV are marked by dashed white lines. (**B-E**) Quantitative analysis of the average signal density in the inner (core) versus outer (shell) ROIs of the tumours. (**B**) The core was defined as the inner region of the tumour demarcated by a spherical surface with half the average radius of the whole tumour, with the shell defined as the rest of the tumour volume. MPI signal voxels were binned into their respective core or shell ROIs, and the average signal density for each ROI was calculated. **(C)** Paired core-shell MPI signal density values for excised OVA$^+$ tumours (n=8). (**D**) Paired core-shell MPI signal density values for excised MC38-OVA tumours (n=8) normalized to the average signal density assessed across entire tumour inclusive of both core and shell regions. (**E**) Correlation plot of MPI signal density values for the core and shell ROI versus tumour mass. Data were analysed using two-tailed ratio-paired t-test, *$p < 0.05$ (**C** and **D**).

Table S1. Details of the labelling reagents used for flow cytometry in this work.

Step	Species reactivity	Fluorophore	Epitope	Clone	Dilution buffer	Dilution	Manufacturer
Live/Dead dye	N/A	Zombie Aqua	Free amines	N/A	PBS	1:500	Biolegend
	N/A	DAPI	dsDNA	N/A	FACS	1:200	Biolegend
Fc receptor blocking	Mouse	N/A	CD16/32	93	FACS	1:50	Biolegend
	Human	N/A	F_c receptors	TruStain FcX	FACS	1:50	Biolegend
Surface epitope staining	Mouse	PE/Dazzle 594	CD4	RM4-5	FACS	1:200	Biolegend
		BV 650	CD8α	53-6.7			
		AlexaFluor 700	CD44	IM7			
		BV 605	CD45.1	A20			
		FITC	CD45.2	104		1:100	
		BV 785	PD-1	29F.1A12		1:200	
		APC-Cy7	TCRβ	H57-597			
		APC	TCR $V_{\alpha2}$	B20.1			
		PE	TCR $V_{\beta5.1,5.2}$	MR9-4			
		BV 421	TIM3	RMT3-23			
	Human	APC-Cy7	CD3	OKT3	FACS	1:200	Biolegend
		PerCP-Cy5.5	CD3	OKT3			
		FITC	CD4	RPA-T4			
		PE/Dazzle 594	CD4	RPA-T4			
		APC-Cy7	CD8α	RPA-T8			
		BV 650	CD8α	RPA-T8			
		APC	CD14	M5E2			
		BV 605	CD19	HIB19			
		PE-Cy7	CD56	MEM-188			

Intracellular epitope staining	Mouse	PE-Cy7	Granzyme B	NGZB	Perm	1:50	Thermo Fisher Scientific
		PerCP-Cy5.5	IFNγ	XMG1.2			Biolegend
		PE	IL-2	JES6-5H4			
		BV 421	T-bet	4B10			
		APC	TNFα	MP6-XT22			
	Human	PE-Cy7	Granzyme B	QA16A02	Perm	1:50	Biolegend
		PerCP-Cy5.5	IFNγ	B27			
		PE	IL-2	MQ1-17H12			
		BV 421	Perforin	B-D48			
		APC	TNFα	W19063E			
